# Illustration of Step-Wise Latent Class Modeling With Covariates and Taxometric Analysis in Research Probing Children's Mental Models in Learning Sciences

**DOI:** 10.3389/fpsyg.2018.00532

**Published:** 2018-04-16

**Authors:** Dimitrios Stamovlasis, George Papageorgiou, Georgios Tsitsipis, Themistoklis Tsikalas, Julie Vaiopoulou

**Affiliations:** ^1^Department of Philosophy and Education, Aristotle University of Thessaloniki, Thessaloniki, Greece; ^2^Department of Primary Education, Democritus University of Thrace, Alexadroupolis, Greece

**Keywords:** latent class analysis, mental models, BCH and ML corrections, taxon, taxometrics MAMBAC, MAXEIG, L-Mode, comparison curve fit index

## Abstract

This paper illustrates two psychometric methods, latent class analysis (LCA) and taxometric analysis (TA) using empirical data from research probing children's mental representation in science learning. LCA is used to obtain a typology based on observed variables and to further investigate how the encountered classes might be related to external variables, where the effectiveness of classification process and the unbiased estimations of parameters become the main concern. In the step-wise LCA, the class membership is assigned and subsequently its relationship with covariates is established. This leading-edge modeling approach suffers from severe downward-biased estimations. The illustration of LCA is focused on alternative bias correction approaches and demonstrates the effect of modal and proportional class-membership assignment along with BCH and ML correction procedures. The illustration of LCA is presented with three covariates, which are psychometric variables operationalizing formal reasoning, divergent thinking and field dependence-independence, respectively. Moreover, taxometric analysis, a method designed to detect the type of the latent structural model, categorical or dimensional, is introduced, along with the relevant basic concepts and tools. TA was applied complementarily in the same data sets to answer the fundamental hypothesis about children's naïve knowledge on the matters under study and it comprises an additional asset in building theory which is fundamental for educational practices. Taxometric analysis provided results that were ambiguous as far as the type of the latent structure. This finding initiates further discussion and sets a problematization within this framework rethinking fundamental assumptions and epistemological issues.

## Introduction

Research on children's mental conceptions of everyday reality, before they acquire the science view, is an interdisciplinary area where psychometrics take the dominate role providing sophisticated tools to access these intangible entities or latent variables in question. By means of mathematical models, probabilistic relations are established between the latent theoretical constructs and their manifestations, which are a set of empirical indicators. The latter are the variables, which, by some means, are *epistemically accessible* to the researcher, while latent variables are not (Borsboom, 2008; p. 28). Their relationship is specified in mathematical terms with a generalized regression function, which comprises a system that provides access to these latent structures, the validly of which, however, is not *a priory* definite. Moreover, the ontology of these latent structures is also undefined and vague. Children's mental representations (knowledge) of physical phenomena are issues investigated in cognitive and developmental psychology, as well as in related educational fields, such as science education. Scholars interested in the nature of children's mental models make relevant assumptions for their ontological statuses, and build theoretical premises that could facilitate interpretations of learning phenomena and suggest the ultimately appropriate teaching methods (Johnson, 1998; Papageorgiou and Johnson, 2005; Taber, 2009). The question of whether the latent variables, i.e., children's mental representations or naïve knowledge of everyday reality, should be treated as discrete entities or as continua is a crucial theoretical entreaty. Similar enquiry subsists in psychopathology research, where, considering latent disorders as continua or discrete kinds has important implications for diagnosis procedures and treatment (Rezai et al., 2010; Edens et al., 2011; Haslam et al., 2012). The challenge at this juncture concerns the measurement theory, which studies such latent variables by means of mathematical structures and suggests the proper formalism and modeling procedures (e.g., Rust and Golombok, 2009; Trendler, 2009). The mathematical structures refer to nominal, ordinal or continuous scales, while measurement implies a categorization process, that is, how to form the equivalence classes. If the children knowledge, reflected by certain mental models, was directly observable, this categorization process would be easy and straightforward. However, even though this does not happen, psychometric theory postulates that this difficulty could be overcome if these latent entities are assumed to be responsible for behaviors that are observable (Markus and Borsboom, 2013). In research on children's mental models the corresponding observable behaviors are item responses, texts or drawings, on which the hypothesized latent entities are conceptualized as the *common cause*. A psychometric model that describes the structure of this common cause is tightly related to a psychological theory expressing it in an explicit way. A theory might specify a hypothesis, that there are *n* different forms of children's response patterns, and thus *n* forms of knowledge, which ontologically are discrete kinds. Thus, a latent class model is the proper representation of the working hypothesis (Clogg, 1995; Dayton, 1998). Making different assumptions about the latent structures in relation to the structure of the observables, one can lead to the known taxonomy of the psychometric models (e.g., factor model, IRT, etc., see Bartholomew, 1987), in which the hypothesized relationships between latent and observable structures could be considered as being analogous to the traditional view of dependent and independent variables, respectively.

The fundamental assumption in all latent structure models is the *principle of local independence*. That is, given a specific level or kind of the latent variable, e.g., a specific mental representation, the empirical indexes are independent conditional on the latent variable, and this is part of the mathematical formalism. The assumption of local independence is associated with the causal interpretation of the variation of the latent on the variation of the observable variables (Bartholomew, 1987; Markus and Borsboom, 2013). It is interesting to mention here that such consideration, epistemologically, adheres to a realist rather than to a constructivist stance and this has been proved appropriate for essentially interpreting psychometric models (Borsboom et al., 2003).

While the distributions of observables are known from the designed data-collection instrument (questionnaire or interview), the distribution of the latent variable is unknown, and thus it might be posited as a research question or hypothesis. When based on theoretical speculations, one implements a certain psychometric model and achieves an adequate fit, this is usually considered as an evidence about the ontological status of the hypothesized latent construct, that is, whether it is a discrete kind or a continuous entity. LCA, when applied, presumes a categorical structure, which could be supported by an adequate model fit.

A complete psychometric modeling, however, should include comparison and selection based on a global best-fit between alternative models in which the latent variable is considered as being categorical and/or as being continuous (e.g., Lubke and Neale, 2006, 2008; Lubke and Miller, 2015). Furthermore, in statistical methods investigating latent structures, psychometrics has made a distinction between the approaches that estimate structural model parameters and those that detect the type of the structural model (McGrath and Walters, 2012). The latter method is called *taxometrics* and is based on examining the consequences of a particular model for the statistical properties, e.g., the covariance among empirical indicators. (Waller and Meehl, 1998; De Boeck et al., 2005; Schmitt et al., 2006; Lubke and Miller, 2015). It has been proposed that the efficient strategy in psychometric research is to apply both approaches in order to avoid misinterpretation of empirical data. In general, taxometrics follows the usual psychometric modeling and comprises a complementary procedure investigating latent constructs. In fields, such as medicine and psychopathology, taxometrics has become a popular method, which is extensively used for uncovering latent causes of various symptoms (e.g., Meehl, 1995; Haslam et al., 2012).

Mental model research, so far, has not incorporated taxometrics as a basic step of analysis, while the fundamental hypothesis on the nature of latent variables in question has a central interest in the relevant theories of knowledge acquisition and conceptual change. Particularly, the nature of the latent variables in question is associated with pathways of development and mental changes, which might be attained in a linear fashion, but also in nonlinear or discontinuous mode (e.g., van der Maas and Molenaar, 1992; Carey, 2009; Stamovlasis et al., 2011; Molenaar et al., 2013). In probing the nature of children's mental representations, LCA has been proved the most suitable approach for obtaining a typology based on a set of observed variables (Straatemeier et al., 2008; Schneider and Hardy, 2013; Stamovlasis et al., 2013; Pluess et al., 2018). By implementing both LCA and TA, a better understanding on the nature of the latent structure can be obtained and the initial assumptions on its ontological status can be justified or questioned. Note that in this inquiry, theoretically at least, two distinct latent classes exist, one of which corresponds to the scientific view. Thus, TA is expected to be in line with LCA assumptions, and this would reinforce relevant theoretical premises and their impact in educational practices. Moreover, if it is desired to investigate how the ensuing latent classes might be related to external variables /covariates, then the effectiveness of classification process and the unbiased estimations of parameters become the central concern.

The present paper illustrates the two types of methods probing latent structures. First, the three-step LCA is presented and applied to two data sets exploring children's mental models of some physical phenomena, with three psychometric variables as covariates operationalizing formal reasoning, divergent thinking and field dependence-independence, respectively. In addition, a third data set corresponding to children's view of the shape of the earth is examined (Panagiotaki et al., 2006; Straatemeier et al., 2008) with age as covariate. The illustration of LCA focuses on alternative bias correction approaches and demonstrates the effect of modal and proportional class-membership assignment along with ML and BCH correction procedure, showing how these estimations can be improved (Bolck et al., 2004; Vermunt, 2010). Moreover, taxometric analysis is applied complementarily to demonstrate the use of such psychometric tool and its potential contribution.

It is emphasized that the input of the present paper is not merely the presentation of LCA in conjunction with TA as an illustrative tutorial. The concurrent implementation of both methods it is proposed here as a strategic framework in investigating children's mental representations ions and it opens a new research avenue. Moreover, the results of both methods are co-examined and theoretical insights based on the empirical findings are discussed.

## Latent class analysis

### Advantages and problems

The merit of latent class analysis (LCA) has been acknowledged from the early stages of its development, due to its sophisticated modeling prospects (e.g., McCutcheon, 1987). Identifying unknown clusters or latent classes, where individuals share identical or alike values is a normal procedure, whereas the foremost research question is to reveal possible causal relationships or consequences of the encountered class-memberships; that is, the association of the latent variable with covariates or distal outcomes. One methodological choice is the one-step LCA, where the relation between latent class-membership and covariates is estimated simultaneously (Yamaguchi, 2000; Muthén, 2004). From a statistical point of view, this approach is preferred when the basic model assumptions hold; otherwise problems might arise in attaining the optimal solution (Tofighi and Enders, 2008; Petras and Masyn, 2010), while additional weak points originate from large number of parameters and/or the sparseness of the frequency tables analyzed (Huang and Bandeen-Roche, 2004; Clark and Muthén, 2009).

The second choice is the step-wise method, which includes three steps: (i) the underlying latent variable is identified based on a set of indicators, (ii) the cases (individuals) are assigned to latent classes, and (iii) the resulted class membership and the covariates are analyzed accordingly (Bolck et al., 2004; Vermunt, 2010). The step-wise approach is preferred when the predictive validity of the covariate is the main concern, while different options in classification procedures can be followed. Nonetheless, the three-step approach has a weak point associated with yielding severely downward-biased estimates of the parameters modeling relationships between class membership and covariates (Bolck et al., 2004; Vermunt, 2010). In the following sections the above issue is illustrated through a formal presentation of LCA and the above correction methods.

### LCA and classification

The LC modeling starts from a basic equation expressing the probability of observing response pattern y defined by:

(1) $$\begin{array}{l} p_{\left(Y=y\right)}=\overset{C}{\underset{c=1}{\sum }}p_{\left(X=c\right)}\times p_{\left(Y=y/X=c\right)} \end{array}$$

Where *X* is the categorical latent variable, *c* is a specific latent class among *C* classes, and *y* is the realization of the vector *Y* measuring the response patterns (X
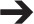
Y). *p*_(X = *c*)_ represents the probability of belonging to class *c* and *p*_(Y = *y*/*X* = *c*)_ the conditional probability of having response pattern *y*, given that *X* belongs to the specific class *c*.

Based on the assumption of local independence and the fact that the joint probability of a specific response pattern on the vector of indicator variables is the product of the item specific probabilities, one arrives at the Equation (2) expressing the probability of observing response pattern as a function of *p*_(X = *c*)_ and the class-specific response probabilities *p*_(_*Y*__*k*_ = *y*_*k*_/*X* = c)_. *K* is the number of mutually independent manifest variables given the class, and *k* = 1, 2,.*K*.

(2) $$p_{\left(Y=y\right)}=\sum_{c=1}^{C}p_{\left(X=c\right)}\times \prod_{k=1}^{K}p_{\begin{array}{c} \left(Y_{k}=y_{k}/X=c\right) \end{array}}$$

The parameters of the model can be estimated by implementation of a maximum likelihood method (ML).

The latent class predictions are made via the posterior probability of belonging to a class *c* given an observed response pattern *y, p*_(X = *c*/*Y* = *y*)_, by applying Bayes's theorem, that is:

(3) $$p_{\left(X=c/Y=y\right)}=\frac{p_{\left(Y=y/X=c\right)}\times p_{\left(X=c\right)}}{p_{\left(Y=y\right)}}$$

Using the above posterior class membership probabilities, it is possible to assign cases to classes, by applying different types of criteria or procedures, the most prevalent of which are the *modal* and the *proportional* assignments (Collins and Lanza, 2010; Bakk et al., 2013). In the modal assignment, each case is assigned to the class with the larger posterior membership probability. If W is the predicted class for a case *i* with response pattern *y*_*i*_, in the modal assignment, a case is assigned with probability equal to the unity, to the class with the largest posterior probability, and with probability zero to the other classes. In mathematical terms:

(4) $$\begin{array}{l} p_{\left(W=s/Y=y_{i}\right)}=1,ifp_{\left(X=s/Y=y_{i}\right)}>p_{\left(X=c/Y=y\right)}\forall s\neq c;\text{and else}\\ \;p_{\left(W=s/Y=y_{i}\right)}=0, \end{array}$$

The modal assignment is considered to be the optimal one, that is, it gives the smallest classification errors. Otherwise.

The proportional assignment follows the so called soft partitioning method (Dias et al., 2008). A case with the response pattern *y*_*i*_, will be assigned to each class *s* (*W* = *s*) with a weight equal to the posterior membership probability [*p*_(*W* = *s*/*Y* = *yi*)_ = *p*_(*X* = *s*/*Y* = *yi*)_].

Regardless of the class-assignment method, always there are cases that are not placed in the right class. The effectiveness of the classification or the *classification error* can be expressed by the probability *p*_(*W* = *s*/*X* = *c*)_, that is the probability of assigning a case to a class conditional to the actual class. If this probability is large for *s* ≠ *c*, then the quality of the classification is problematic. The overall proportion of misclassifications can be obtained by taking the average misclassification probabilities of all empirical data patterns, and an *overall classification error* (CE) can be calculated. Thus, the classification errors are:

(5) $$\begin{array}{l} p_{\left(W=s/X=c\right)}=\frac{\frac{1}{N}\overset{N}{\underset{i}{\sum }}p_{\left(X=c/Y=y_{i}\right)}w_{is}}{p_{\left(X=c\right)}} \end{array}$$

where *N* is the sample size and *w*_*is*_ = *p*(*W*_=*s*_/*Y*_=*y*_*i*__).

A similar concept to classification error (CE) is the concept of *separation between classes* (SBC). SBC refers to how well the classes can be distinguished based on the existing empirical information. It is obvious that lower SBC corresponds to larger CE. A measure for class separation, and thus also for CE, can be the expression of how much the posterior membership probabilities *p*_(*X*_=*s*_/*Y*_=*y*_*i*__)_ deviate from uniform. Vermunt and Magidson (2005) proposed the use of entropy measures:

(6) $$\begin{array}{l} I=\overset{C}{\underset{c=1}{\sum }}\left[P_{\left(X=c/Y=y\right)}\times \log P_{\left(X=c/Y=y\right)}\right] \end{array}$$

The quality of classification, expressing how well the classes are separated, can be measured by the *(entropy) pseudo R*^2^, which is defined as the proportional change of entropy *I*, when *Y* is available compared to the case in which *Y* is unknown (Vermunt and Magidson, 2005).

### Introduction of covariates

**Figure 1:**
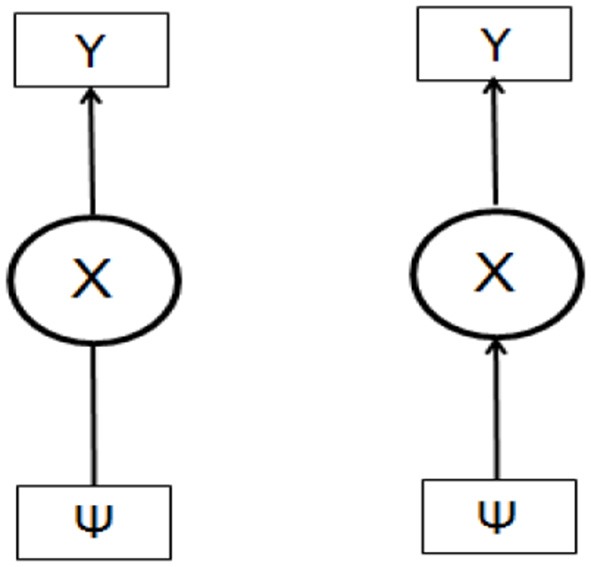
The LC model with covariate (a general scheme).

Figure 1 (left side) depicts a LC model in a more general form, where the latent variable *X* is measured by *Y* (vector of indicators) and no specific causal relationship between *X* and the external variable(s) Ψ is stated. In Figure 1 (right side) the covariate Ψ is assigned in the role of predictor variable of the latent construct *X*.

This modeling encompasses the joint probability of the three sets of variables (*X, Y*, and Ψ):

(7) $$p_{\left(\Psi =\psi ,X=c,Y=y\right)}=p_{\left(\Psi =\psi ,X=c\right)}p_{\left(Y=y/X=c\right)}$$

Assuming the principle of local independence for *Y* and Ψ, given *X*, and including the condition that the latent variable depends on the covariate Ψ, the relation Equation (8) can be finally stated from which the relationship between *X* and Ψ can be analyzed:

(8) $$\begin{array}{l} p_{\left(X=c,Y=y/\text{Ψ}=\psi \right)}=p_{\left(X=c/\text{Ψ}=\psi \right)}p_{\left(Y=y/X=c\right)} \end{array}$$

The above model needs the specification of the conditional distribution (normal, ordinal or nominal) of Ψ in order to quantify its relations of Ψ-*Y*, while the corresponding regression model would be linear, cumulative logistic and multinomial logistic regression, respectively (Bakk et al., 2013).

### The step-wise approach

**Figure 2:**
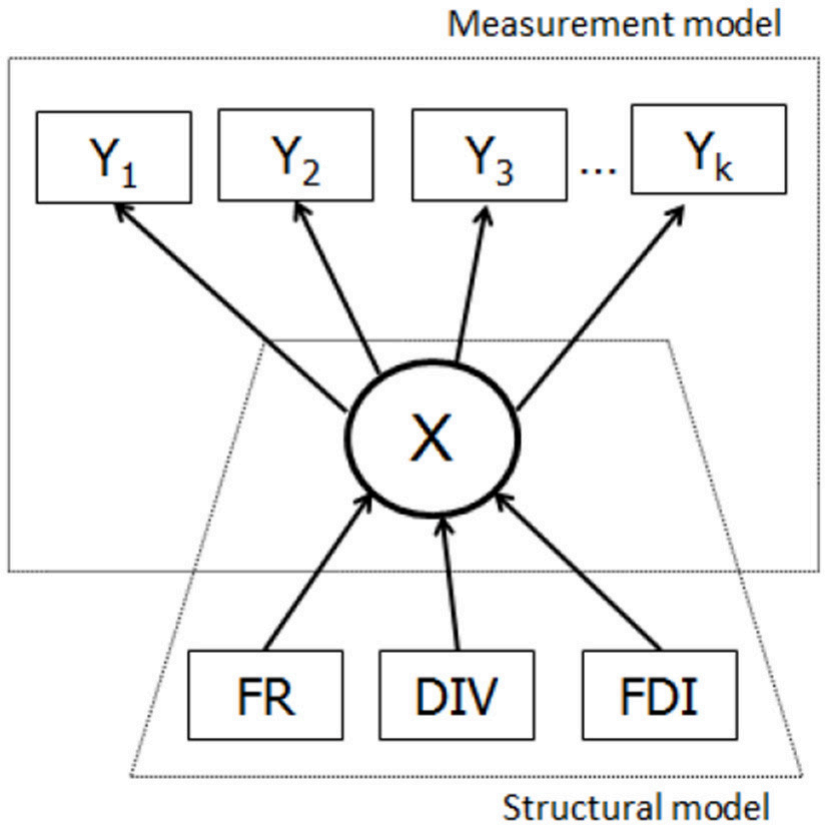
The latent variable model with the three covariates.

Figure 2 shows schematically the traditional one step LCA, where the covariates are incorporated in the latent class model which can be seen as being composed of two parts: the measurement model that includes information on indicators Y_1_, Y_1_ Y_k_ given X and the structural part that deals with the relationship between X and covariate(s) Ψ (FR, DIV & FDI).

**Figure 3:**
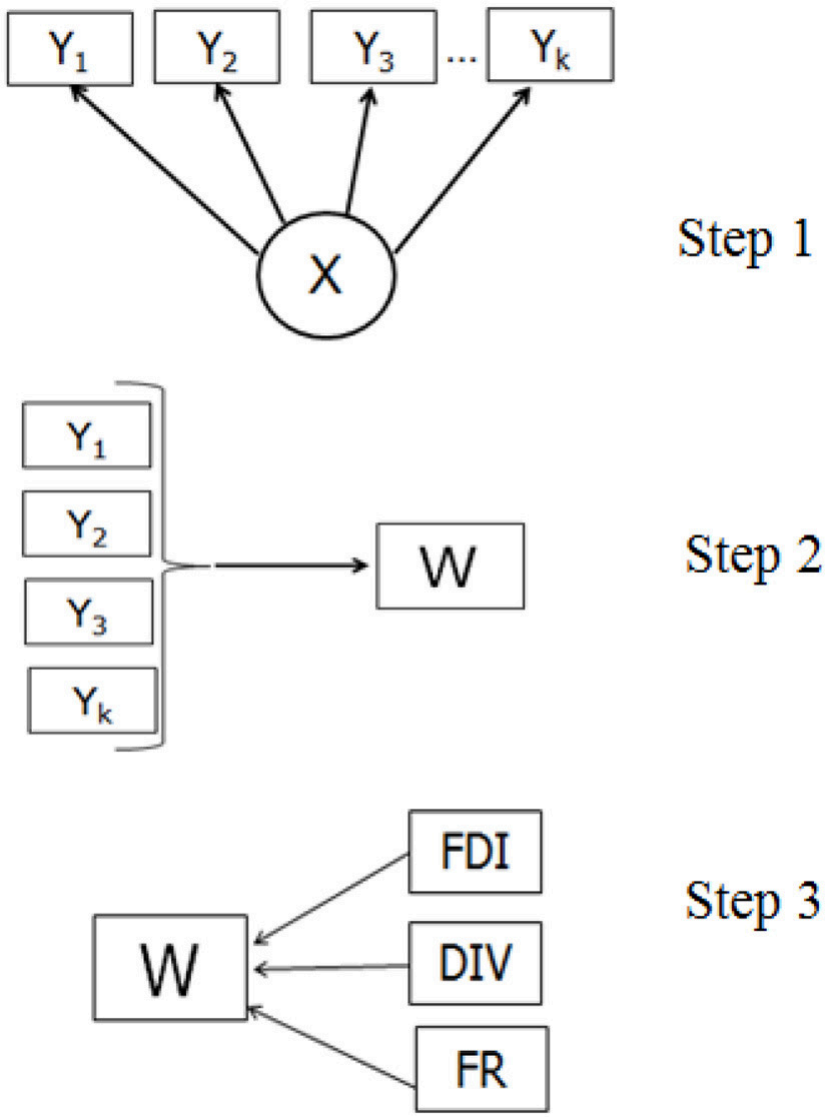
The three steps of the step-wise latent class analysis with the three covariates.

The three-step approach (Figure 3) proceeds with the measurement model, building the relationships between the latent variable and its indicators: X 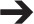 Y (Y_1_, Y_1_. Y_k_). In the next step, based on the information from the previous step, the cases are assigned to latent classes based on scores measured with indicator variables (Y 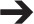 W). Different assignment rules can be used, such as the modal or proportional assignment. In the third step, the predicted class membership variable (W) is used to establish the relationship between W and Ψ (FR, DIV, & FDI). In the standard three-step procedure, although the relationship between W and Ψ is estimated, the target is the relationship between X and Ψ. Bolck et al. (2004) proved that the estimates of the log-odds ratios characterizing the relationship between Ψ and W will always be smaller than those characterizing the relationship between Ψ and X. A correction method needs to reveal first the relationship between the distributions of X-Ψ and W-Ψ. Given that W depends only on Y (because the classification was obtained in this way), and the Y is independent of Ψ given X, it was shown that the entries in the W and Ψ distribution are weighted sums of the entries in the X and Ψ distribution, where the weights are the misclassification probabilities *p*_(*W* = *s*/*X* = *c*)_. Thus, these misclassification probabilities can be used to correct the W - Ψ relationship to get the relationship between X and Ψ (Bolck et al., 2004).

A correction approach (BCH) developed by Bolck et al. (2004) involves re-expressing the relationship describing the *p*_(*W* = *s*, Ψ = ψ)_ and uses the weighted W-Ψ distribution by the inverse of the classification errors (Bolck et al., 2004), which applies at the population level, to reweight the data on W and Ψ. This approach involves maximizing a weighted long-likelihood function, where the reweighted frequencies are used to estimate the relations between X and Ψ (Vermunt, 2010). The disadvantages of this method are that, it holds only for categorical external predictor variables, the SEs are underestimated, while the method needs a tedious data preparation stage.

Vermunt (2010) in order to solve these issues, proposed a modification to the BCH method focusing on re-expressing the pseudo log likelihood function in terms of individual observations. That is,

(9) $$\begin{array}{l} LogL_{BCH}=\sum_{i}^{N}\sum_{s}^{W}w_{is}\sum_{c}^{C}d_{sc}^{*}\log p_{\left(X=c,\Psi =\psi_{i}\right)}\\ \;=\sum_{i}^{N}\sum_{c}^{C}w_{ic}^{*}\log p_{\left(X=c,\Psi =\psi_{i}\right)} \end{array}$$

where the *w*_*is*_ is a class assignment weight, $d_{\text{sc}}^{*}$ is an element in the inverted matrix of the probabilities *p*_(*W* = *s*/*X* = *c*)_ and *w*_*ic*__=_ Σw_ι*s*_ $d_{\text{sc}.}^{*}$

This weighted data set can be analyzed with standard methods. While the above equation shows how to estimate parameters of the joint distribution of *X* and Ψ, it can be modified for the estimation of the conditional distribution of Ψ given *X* (Bakk et al., 2013). Note that this formulation makes it possible to apply the BCH method to external variables of any scale type. Moreover, it applies a robust or sandwich variance estimator and prevents the underestimation of SEs' (as in the original BCH approach). The ML-based method introduced by Vermunt (2010) differs from the standard LCA in that the conditional responses probabilities are fixed to values estimated in the previous step.

## Materials and methods

LCA was applied to data originated from three empirical studies probing children's mental representations in science learning. Moreover, in the first two studies three covariates were used as independent variables predicting the latent variable: Formal reasoning (FR), divergent thinking (DIV) and field dependence-independence (FDI) respectively. In the third study, age was treated as covariate predicting class membership. Data analysis and parameter estimation were carried out using Latent GOLD version 5.1 (Vermunt and Magidson, 2008).

### Data and procedures

#### Study 1

##### Sample

Participants were 329 ninth-grade junior high school Greek students belonging to 18 classrooms from different schools located in the area of central Greece. The age of the participants was 14–15, 52% of which were female and 48% male and they were of different socioeconomic status and living conditions.

##### Measurements

The instrument used for data collection was a questionnaire measuring the understanding of the particulate nature of matter and its changes of states (melting, boiling, condensation and evaporation). The instrument included illustrations and questions measure at ordinal scale, which were valid empirical indicators for assessing students' conceptual understanding on this matter, designed and implemented in a series of studies (e.g., Johnson, 1998; Papageorgiou et al., 2010; Tsitsipis et al., 2010). For the present sample, the Cronbach's alpha reliability coefficient was 0.86, while further validity issues could be found elsewhere (e.g., Stamovlasis et al., 2012). In addition, students were tested for the three psychometric variables (see below).

#### Study 2

##### Sample

The study was conducted with the participation of 375 sixth-grade primary school Greek pupils (age 11–12, 49.1% females). The participants were of different socioeconomic status and attended 17 different schools in Northern Greece. All subjects had been taught an introductory course in physical science, according to the curriculum, during the previous academic year.

##### Measurements

Students were tested for their understanding of the particulate nature and the changes of state of matter. A simplified version of the questionnaire from study 1, adapted to the age of the participants was used (Tsitsipis et al., 2010; Stamovlasis et al., 2012). Cronbach's a reliability coefficient of the instrument was 0.79.

### Covariates

The participants were also assessed for the following psychometric variables:

#### Field dependence/independence (FDI)

FDI ability of the subjects was assessed by a version of the Witkin et al. (1971) Group Embedded Figures Test (GEFT). Cronbach's alpha reliability coefficient ranged 0.84. The scale treated as uni-dimensional demonstrated a good fit using CFA [$\chi_{\left(152\right)}^{2}$ = 302.6, *p* < 0.001, CFI = 0.98, TLI = 0.98, RMSEA = 0.049 (0.042–0.057)].

#### Divergent thinking (DIV)

DIV was measured by a special test, which has been extensively used in science education research for measuring divergency of also Greek students (Danili and Reid, 2006; Stamovlasis et al., 2012). The test comprises thinking, generating or constructing as many items as possible, including words, sentences, drawings or ideas having an opposite or common trait. Cronbach's alpha reliability coefficient of the instrument was 0.76. Indicatively, a unidimensional CFA model demonstrated good fit [$\chi_{\left(82\right)}^{2}$ = 112.2, *p* = 0.07, CFI = 0.99, TLI = 0.99, RMSEA = 0.029 (0.000–0.045)].

#### Formal reasoning (FR)

Pupils' logical thinking abilities were measured with the Lawson paper-and-pencil test of formal reasoning (Lawson, 1978, 1983). The test consists of 15 items involving the following: conservation of weight, displaced volume, control of variables, proportional reasoning, combinational reasoning and probabilistic reasoning. Cronbach's alpha reliability coefficients were in the range of 0.79. Indicatively, a unidimensional CFA model demonstrated good fit [$\chi_{\left(84\right)}^{2}$ = 103.4, *p* = 0.06, CFI = 0.99, TLI = 0.99, RMSEA = 0.026 (0.000–0.039)].

#### Study 3

##### Sample

Participants were 502 elementary school Greek students belonging to 15 classrooms from different schools located in the area of central and northern Greece. The age of the participants was from 6 to 12 years old [6–7 (18.3%), 7–8 (12.4%), 8–9 (19.9%), 9–10(21.7%) 10–11 (14.1%), and 11–12 (13.5%)], 50.2% of which were female and 49.8% male, and they were of different socioeconomic status and living conditions.

##### Measurements

Children were tested via a close-ended questionnaire concerning the shape of the earth and related phenomena. The instrument was the EARH questionnaire, designed and used in previous research (Straatemeier et al., 2008). It is a structured, nonverbal, forced-choice test that can be easily administered, while training of experimenters and the use of complex coding systems are not required. The EARTH includes a number of illustrations, which describe the most prevalent models found in earlier studies with samples from Western countries (Vosniadou and Brewer, 1992; Vosniadou, 1994), while an ordinal marking scheme was used for the purpose of the present analysis. The participants have to make only one choice by examining the pictures and decide which of them fits better to what they have in mind. The questionnaire is freely available on the internet (Straatemeier et al., 2008).

## Results

The step-wise LCA with covariates (Figures 3) was applied. The correction methods of BCH and ML were used and the parameter estimates and the corresponding standard deviations of the covariates are presented. The one-step LCA besides the disadvantages mentioned in a preceding section (Vermunt, 2010) has a weak point associated with the external variables. When the distributions of the covariates strongly deviate from normality, the latent class formation can be highly distorted (Asparouhov and Muthén, 2013). This holds for the covariates of the present model, however, results from the one-step LCA are presented merely for comparison reasons.

### Results of study 1

The first step was to identify the number of classes representing distinct groups of students possessing certain mental representation on the basis of nine items regarding the structure of matter. From the analysis, the two-class (entropy *R*^2^ = 0.89, *p* = 0.16, classification error = 0.026, BIC = 7011.7, Npar = 75) and the three-class (entropy *R*^2^ = 0.78, *p* = 0.24, classification error = 0.088, BIC = 6945.5, Npar = 85) solutions were the best parsimonious models in terms of entropy *R*^2^, BIC, and *p*-values. However, conditional bootstrap test of L^2^(3-class) - L^2^(2-class) showed that the three-class solution did not provide any significant improvement, so the two-class solution was chosen based on entropy measures, classification errors and parsimony. The classes corresponded to 25.7% and 74.3% of the sample respectively. Class 1 includes high achievers, that is, those students who attained the scientific view, whereas Class 2 includes low achievers, students who did not possess a clear understanding of the subject matter. Results concerning Class 1 are presented in Table 1, showing the effects and standard errors of the three covariates on class membership. It is observed that the standard proportional and modal classification give the lowest values, because of the aforementioned downward-biases estimation (Bolck et al., 2004), while the use of BCH and ML corrections methods adjusts these parameter values.

**Table 1:** Effects, *Z*-values and standard errors of covariates, formal reasoning (FR), divergent thinking (DIV) and field dependence-independence (FDI) on class membership, Class 1 in Study 1.

	**FR**			**DIV**			**FDI**		
	**Mean**	**s.e**.	***Z*-value**	**Mean**	**s.e**.	***Z*-value**	**Mean**	**s.e**.	***Z*-value**
One-step ML	0.092	0.015	6.273	0.043	0.014	3.101	0.056	0.029	1.914
Proportional	0.075	0.011	6.742	0.029	0.010	2.954	0.037	0.022	1.695
Proportional ML	0.097	0.016	5.878	0.036	0.011	3.190	0.046	0.024	1.880
Proportional BCH	0.102	0.019	5.504	0.044	0.014	3.134	0.055	0.029	1.909
Modal	0.074	0.011	6.750	0.029	0.010	3.003	0.032	0.022	1.460
Modal ML	0.088	0.016	5.668	0.034	0.012	2.959	0.035	0.025	1.374
Modal BCH	0.089	0.016	5.686	0.037	0.013	2.927	0.041	0.027	1.497

### Results of study 2

The identification of the number of classes representing distinct groups of children possessing certain mental representation was based on seven items regarding physical phenomena. From the analysis, the 3-class solution (entropy *R*^2^ = 0.73, *p* = 0.20, classification error = 0.091) was chosen based on BIC values. The latent classes were: Class 1 (with 17.00%), Class 2 (with 68.85%) and Class 3 (with 14.15%), which correspond to low, intermediate and high achievement students respectively. Tables 2.1–2.3 show the effects and standard errors of the three covariates on class membership for Classes 1, 2, and 3 respectively, where the differences among the correction methods can be observed. The effects and standard deviations of the three covariates are smaller for standard proportional and modal classification due to the downward-biases estimation, while correction is achieved by the BCH and ML methods (Bakk et al., 2013).

**Table 2.1:** Effects, *Z*-values and standard errors of covariates, formal reasoning (FR), divergent thinking (DIV) and field dependence-independence (FDI) on class membership, Class 1 in Study 2.

	**FR**			**DIV**			**FDI**		
	**Mean**	**s.e**.	***Z*-value**	**Mean**	**s.e**.	***Z*-value**	**Mean**	**s.e**.	***Z*-value**
One-step ML	0.223	0.041	5.410	0.094	0.021	4.519	−0.018	0.054	−0.326
Proportional	0.111	0.022	5.010	0.038	0.010	3.810	−0.026	0.032	−0.809
Proportional ML	0.194	0.033	5.855	0.069	0.021	3.375	−0.047	0.046	−1.028
Proportional BCH	0.260	0.073	3.537	0.092	0.032	2.907	−0.059	0.057	−1.048
Modal	0.149	0.024	6.087	0.046	0.011	4.193	−0.058	0.033	−1.749
Modal ML	0.206	0.040	5.199	0.068	0.024	2.839	−0.085	0.053	−1.600
Modal BCH	0.249	0.067	3.730	0.080	0.029	2.722	−0.098	0.061	−1.599

**Table 2.2:** Effects, *Z*-values and standard errors of covariates, formal reasoning (FR), divergent thinking (DIV) and field dependence-independence (FDI) on class membership, Class 2 in Study 2.

	**FR**			**DIV**			**FDI**		
	**Mean**	**s.e**.	***Z*-value**	**Mean**	**s.e**.	***Z*-value**	**Mean**	**s.e**.	***Z*-value**
One-step ML	0.019	0.026	0.722	−0.025	0.012	−2.062	−0.026	0.034	−0.765
Proportional	0.007	0.014	0.486	−0.008	0.006	−1.303	−0.008	0.021	−0.399
Proportional ML	0.016	0.022	0.740	−0.016	0.012	−1.301	−0.009	0.030	−0.283
Proportional BCH	−0.008	0.043	−0.179	−0.026	0.018	−1.489	−0.014	0.037	−0.385
Modal	0.005	0.015	0.311	−0.014	0.007	−2.082	0.008	0.021	0.370
Modal ML	0.008	0.025	0.298	−0.023	0.014	−1.664	0.021	0.034	0.631
Modal BCH	−0.002	0.039	−0.051	−0.027	0.016	−1.632	0.016	0.039	0.418

**Table 2.3:** Effects, *Z*-values and standard errors of covariates, formal reasoning (FR), divergent thinking (DIV) and field dependence-independence (FDI) on class membership, Class 3 in Study 2.

	**FR**			**DIV**			**FDI**		
	**Mean**	**s.e**.	***Z*-value**	**Mean**	**s.e**.	***Z*-value**	**Mean**	**s.e**.	***Z*-value**
One-step ML	−0.242	0.040	−5.999	−0.069	0.016	−4.219	0.043	0.048	0.909
Proportional	−0.118	0.022	−5.438	−0.030	0.009	−3.370	0.035	0.029	1.198
Proportional ML	−0.210	0.033	−6.332	−0.054	0.017	−3.159	0.056	0.043	1.304
Proportional BCH	−0.252	0.052	−4.886	−0.066	0.020	−3.375	0.073	0.047	1.580
Modal	−0.154	0.023	−6.719	−0.032	0.010	−3.333	0.050	0.030	1.652
Modal ML	−0.214	0.037	−5.803	−0.045	0.018	−2.480	0.064	0.046	1.371
Modal BCH	−0.247	0.051	−4.851	−0.053	0.019	−2.766	0.082	0.049	1.661

### Results of study 3

LCA analysis was based on nine items/questions regarding the shape of the earth and related phenomena. From the analysis the 3-class solution was chosen based on entropy measures (entropy *R*^2^ = 0.79, classification error = 0.086). The latent classes were: Class 1 (with 31.1%), Class 2 (with 50.81%) and Class 3 (with 18.09%), which correspond to high, intermediate and low achievement students respectively. Tables 3 shows the results for Classes 1, 2, and 3, where the difference among the correction methods can be observed. The effects and standard deviations of the *Age* as covariate are smaller for standard proportional and modal classification due to the downward-biases estimation, while correction is achieved by the BCH and ML methods (Bakk et al., 2013).

**Table 3:** Effects, Z-values and standard errors of covariate Age on class membership, in the three Classes in Study 3.

	**Class 1**			**Class 2**			**Class 3**		
	**Mean**	**s.e**.	***Z*-value**	**Mean**	**s.e**.	***Z*-value**	**Mean**	**s.e**.	***Z*-value**
One-step ML	0.5306	0.0675	7.8585	−0.2381	0.0540	−4.4054	−0.2925	0.0671	−4.3616
Proportional	0.4142	0.0516	8.0312	−0.1655	0.0425	−3.8959	−0.2487	0.0558	−4.4573
Proportional ML	0.5030	0.0617	8.1557	−0.2218	0.0534	−4.1552	−0.2812	0.0673	−4.1781
Proportional BCH	0.5182	0.0656	7.8968	−0.2344	0.0546	−4.296	−0.2838	0.0663	−4.2828
Modal	0.3998	0.0508	7.8755	−0.1837	0.0426	−4.3162	−0.2161	0.0544	−3.9728
Modal ML	0.4614	0.062	7.4433	−0.2234	0.0545	−4.0978	−0.238	0.0644	−3.6967
Modal BCH	0.4663	0.064	7.2835	−0.227	0.0554	−4.0977	−0.2393	0.0643	−3.7199

## Taxometric analysis

### General

*Taxometric* analysis (TA) is a statistical method designed to test whether a latent construct, measured by a number of ordinal or continuous observed variables, is dimensional (continuous) or categorical (named as *taxon)*. The idea of taxometrics belongs to Paul Meehl and his co-workers, who developed a procedure for determining whether observed variations are underpropped by a discrete latent cause or taxon (Meehl, 1995). The option of inferring the existence of taxa and discriminating them from latent dimensional variables has tremendous implications in psychological sciences because it affects how individual differences, traits or attributes should be conceptualized, defined, measured and interpreted. A taxon is conceptualized as a pure category with distinct boundaries, while a non-taxonic case needs a conventional diagnostic threshold on an external manifest variable. The fundamental inquiry that motivates taxometrics is common to all psychological endeavors and it has been more influential in fields such as clinical psychology, personality and antisocial behavior research (Walters, 2011), and to psychiatry, where the *taxon* hypothesis has a direct impact on tool development, classification and diagnosis (e.g. DSM-V; Widiger and Samuel, 2005). TA has been used extensively in probing psychopathologies, such as addictions, schizotypy and autistic disorders (Cuesta et al., 2007; Rawlings et al., 2008; James et al., 2016).

Methodologically, TA does not enact a particular structure in the data, e.g., a categorical structure or a set of underlying dimensions; instead it carries out comparison tests between these two alternatives. It differs also from other approaches, because it does not use a single method, i.e., a statistical test. It implements a multiple mathematical procedures and makes decisions based on the consistency among findings. This approach was referred as *coherent cut kinetic* (Meehl, 1995) and it is based on both numerical outputs and interpretations of graphical representations (Ruscio et al., 2006; McGrath and Walters, 2012). TA is implemented complementarily with other psychometric approaches and if it is used systematically, it can shed light into the nature of latent constructs, which might be varied with the sample type, age, lifestyle and even cultural differences (e.g., Fiske, 2002; Walters, 2007).

Taxometrics does not necessarily precede to other statistical analyses and it is essentially worth carrying out if a number of criteria are encountered (Lenzenweger, 2004): First, there is a significant model under study, which indeed implies latent taxon; second, there is a sound underlying theoretical premise, and third, the identification of taxon would strongly affect the conceptualization, the assessment and the treatment of that latent variable. Thus, taxometrics is apt for mental model research, where the above criteria are met and the scientific taxonomy needs a sound methodological and empirical foundation.

### Taxometric graphs

The basic idea in taxometrics is the use of a cut-off diagnosis or base rate. The procedure starts by assigning one of the variables as the *input* variable, while the others are named *output* variables. Based on scores all cases are sorted along the input variable and they partitioned into “cuts” (“windows”). Then, statistical operations are performed on the output variables from where it is possible to get information about the latent structure under examination (Ruscio et al., 2006). In the next sections the most common operations and the resulted measures are briefly described, while detailed and lucid presentations could be found elsewhere (Meehl and Yonce, 1994; Ruscio et al., 2006).

### MAMBAC

In the *Mean Above-Minus Below A Cut* (MAMBAC) the input variable is sorted and a series of cut-points are located along it. Then, on the output variable, the mean difference for scores being above and below each cutting score is calculated. The MAMBAC plot depicts these mean difference series in y axis, while x axis represents the sorted case numbers. In prototypical categorical data, peaks appear near the cutting scores, while in prototypical dimensional data, the plot takes “bowl-shaped” form (Meehl and Yonce, 1994). With number of variables k ≥ 2, all possible input-output pairings are examined and k(k – 1) analyses are performed.

### MAXCOV

The *Maximum Covariance* (MAXCOV) procedure involves one input and two output variables. Within the ordered subsamples defined by the cuts, the covariance of the output variables is calculated. In the MAXCOV graph, the covariance is plotted along the y axis as a function of the mean scores of the input indicator plotted on the x axis (Meehl and Yonce, 1996). The graph actually shows how the covariance between the two indicators changes with the levels of the input indicator. Prototypical categorical data demonstrate peaked curves with a maximum value within the subsamples, while prototypical dimensional data show flat curves, because the covariance remains relatively constant because of the shared loadings on the hypothetical latent dimension. When the analysis is based on number of indicators k > 3, they are examined in triplets, which finally produces k(k − 1)(k − 2)/2 MAXCOV curves.

### MAXEIG

In the *Maximum Eigenvalue* (MAXEIG), instead of calculating the covariance between two output variables, the largest eigenvalue of the covariance matrix is used (Waller and Meehl, 1998), so it includes all available indicators in one step analysis; starting with one indicator as input indicator and the other k-1 indicators as output indicators, it produces k MAXEIG curves. The algorithm for MAXEIG curves and their interpretation are similar to MAXCOV case (Ruscio et al., 2006).

### L-Mode

The *L-Mode* (Latent Mode) procedure is based on a factor analysis and it requires at least three observable variables. For the first factor, factor scores are calculated using Bartlett's weighted least squares method, and their frequency distribution curve is examined. Bimodality is expected if the data are categorical data, while the mode locations can be used to estimate the taxon base rates. On the contrary, the dimensional data exhibit unimodal distributions (Waller and Meehl, 1998; Walters et al., 2010).

**Figure 4:**
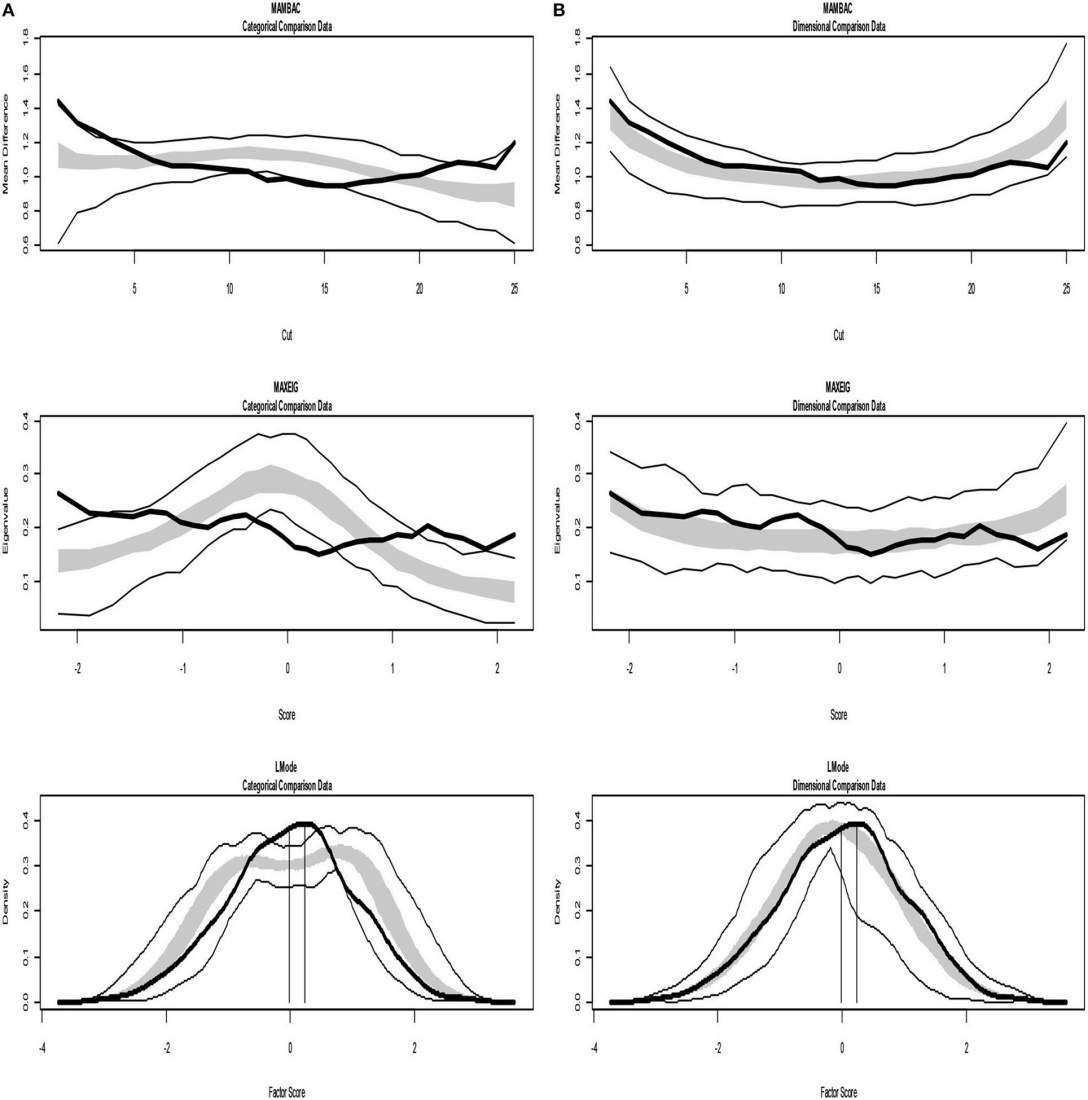
Input: Artificial continuous data. **(A)** Comparison with categorical data. **(B)** Comparison with dimensional data. Results for MAMBAC (top), MAXEIG (middle), and L-Mode (bottom) analyses. Dark lines show the results for prototypical dimensional data, and lighter lines show the results for parallel analyses of comparison data; the lines contain a band that spans ±1 SD from the mean at each data point on the curve.

**Figure 5:**
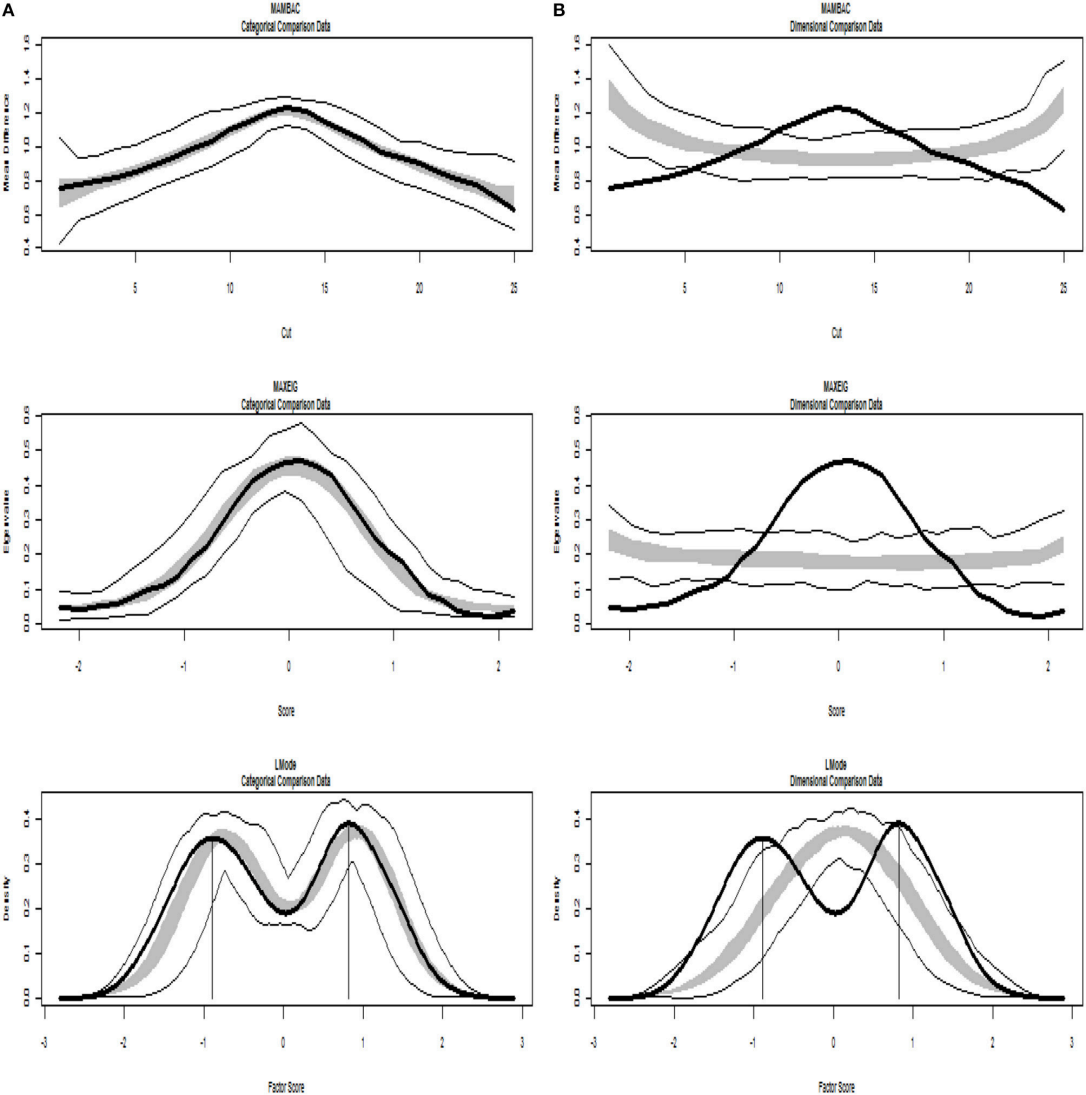
Input: Artificial categorical data. **(A)** Comparison with categorical data. **(B)** Comparison with dimensional data. Results for MAMBAC (top), MAXEIG (middle), and L-Mode (bottom) analyses. Dark lines show the results for prototypical categorical data, and lighter lines show the results for parallel analyses of comparison data; the lines contain a band that spans ±1 SD from the mean at each data point on the curve.

Figures 4, 5 present TA analysis of artificial data with known dimensional and taxon latent structures respectively, which were used as input data (instead of empirical data) in order to demonstrate the interpretations of the taxometric graphs. Both figures show MAMBAC (top), MAXEIG (middle), and L-Mode (bottom) analyses. Dark lines show the results for input artificial data, dimensional or categorical, and lighter lines show the results for parallel analyses of comparison data acquired through simulations techniques; the lines contain a band that spans ±1 SD from the mean at each data point on the curve.

### The comparison curve fit index (CCFI)

As described earlier, taxometric analysis provides plots revealing irregularities, such as discontinuities or distinct peaks that suggest taxon latent variables. The dominate technique in TA is based on comparisons of graphs using parallel analyses with simulated data, which reproduced important characteristics of the empirical data, such as number of variables, sample size, marginal distributions and correlation matrices. Monte Carlo studies have provided strong evidences for the validity and robustness of simulated data techniques (Ruscio and Kaczetow, 2009). The findings of taxometric analysis are interpreted always via comparisons of the real/empirical data with bootstrapped datasets representing idealized categorical and dimensional structures and provide a quantitative fit-index of the two competing models. The key measure is the *Comparison Curve Fit Index* (CCFI), which reflexes the degree to which the results (graphical representations) for the empirical data under investigation are matching to the simulated comparison data, categorical and/or dimensional. The calculation of CCFI involves the *Root Mean Square Residual* (RMSR) defined as:

(10) $$RMSR_{cat}={\left[\sum {\left(y_{emp}-y_{cat}\right)}^{2}/N\right]}^{1/2}$$

Where *N* is the number of data point in the graph and (*y*_*emp*_ – *y*_*cat*_) the distance between points for the empirical data (*y*_*emp*_) and the corresponding points for the categorical comparison data (*y*_*cat*_). The value of *RMSR*_*cat*_ = 0 denotes perfect fit. Analogous calculations are made for *RMSR*_dim_ corresponding to dimensional data.

Then, CCFI is obtained by the equation:

(11) $$\begin{array}{l} CCFI=\frac{RMSR_{\dim}}{\left(RMSR_{\dim}+RMSR_{cat}\right)} \end{array}$$

The values of CCFI index range between 0 and 1. A CCFI > 0.5 suggests a categorical structure or taxon, in the observed data, while a CCFI < 0.5 suggests dimensional structure. The use of the CCFI reduces, to some extent, the threat of confirmation bias in the interpretation of taxometric results (Ruscio and Kaczetow, 2009; Simmons et al., 2011). It is possible to calculate the standard error of the CCFI and defined with increased accuracy the ambiguous findings (Ruscio et al., 2017). A conservative choice is to consider cases in the zone 0.4 < CCFI < 0.6 as ambiguous. In the procedure of calculating CCFI, it is preferable to average curves than to average the estimated CCFIs (Ruscio et al., 2017).

### Outline of taxometric procedure

Taxometric analysis entails an iterative process of multiple steps where the comparison curve fit index and interpretations of the taxometric graphs are implemented (Ruscio and Kaczetow, 2009; Ruscio et al., 2017). The CCFIs are computed as follows:

*First step*: The usual curves are produced and if there are multiple, the average is computed.
*Second step*: Two comparison populations are generated by bootstrapping the empirical data. One population is created under the assumption of categorical and the other under the assumption of dimensional structures respectively.
*Third step*: Random sample from the two artificial populations are drawn and they introduced to taxometric analysis. For both cases, averages of the generated curves are computed.
*Fourth step*: The root-mean-squared residuals (RMSR) between the mean curves of the generated data and empirical data are calculated.
*Fifth step*: The CCFI is calculated via the Equation (11). Decisions are made on the basis of values >0.5 for latent taxon or values < 0.5 for dimensional latent structure.

More information and further mathematical details could be found in Ruscio et al. (2017). It should be pointed out that even though the involved calculations are multiple and complicated, performing taxometric analysis is not a difficult task. It can be easily carried out in R via the *RTaxometrics* package. In Appendix A, the syntax in R used in the present analyses is provided as a guide, which can be applied to analogous data sets.

### Taxometric analysis of the empirical data

Before running taxometric analysis, data should be checked for meeting certain requirements, such as sample size (*N* ≥ 300), number of variables (*k* ≥ 2), number of ordered categories per variable (*C* ≥ 4), between-group validity of each variable (*d* ≥ 1.25), or within-group correlations among variables (*r* ≤ 0.30) (Meehl, 1995; Ruscio et al., 2010). For the present samples, most of the requirements were met. However there were a few violations and borderline values, which might have an impact on the results. However, a number of simulation studies have shown that they could be counterbalance by particularly satisfactory values on other criteria in the same data (Ruscio et al., 2011).

**Figure 6:**
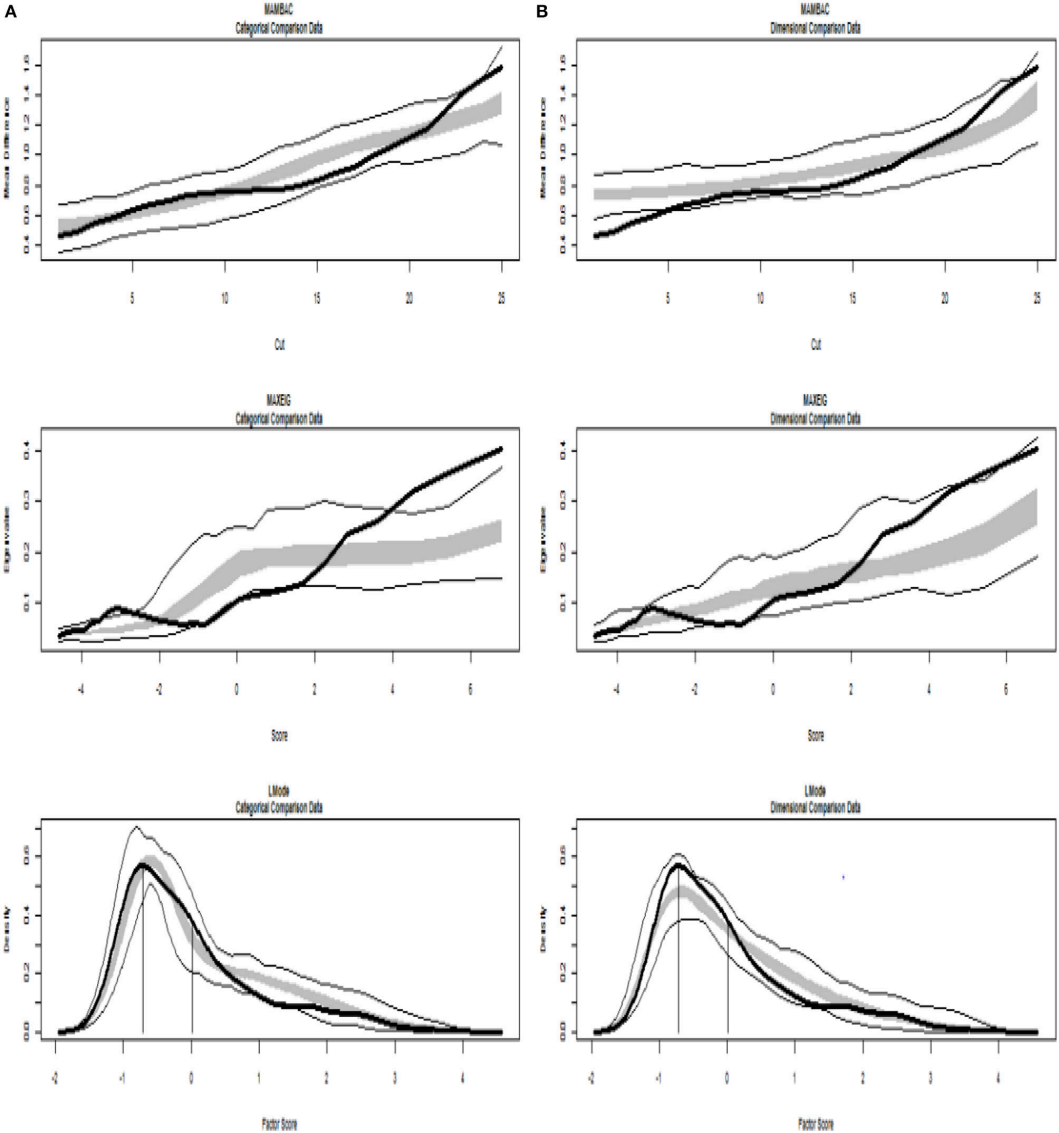
Input: Empirical data – study 1. **(A)** Comparison with categorical data. **(B)** Comparison with dimensional data. Results for MAMBAC (top), MAXEIG (middle), and L-Mode (bottom) analyses. Dark lines show the results for empirical data, and lighter lines show the results for parallel analyses of comparison data; the lines contain a band that spans ± 1 SD from the mean at each data point on the curve.

**Figure 7:**
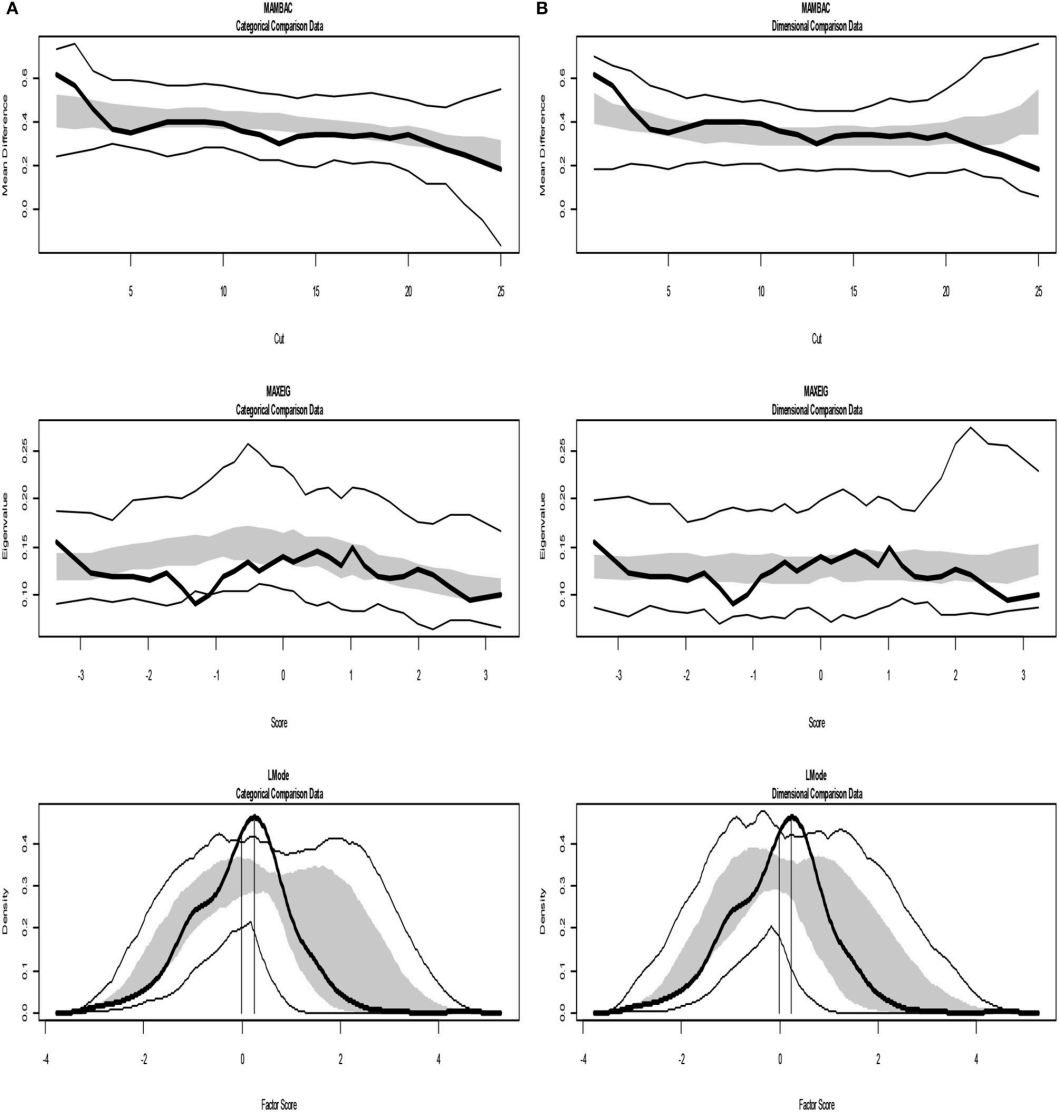
Input: Empirical data –study 2. **(A)** Comparison with categorical data. **(B)** Comparison with dimensional data. Results for MAMBAC (top), MAXEIG (middle), and L-Mode (bottom) analyses. Dark lines show the results for empirical data, and lighter lines show the results for parallel analyses of comparison data; the lines contain a band that spans ±1 SD from the mean at each data point on the curve.

**Figure 8:**
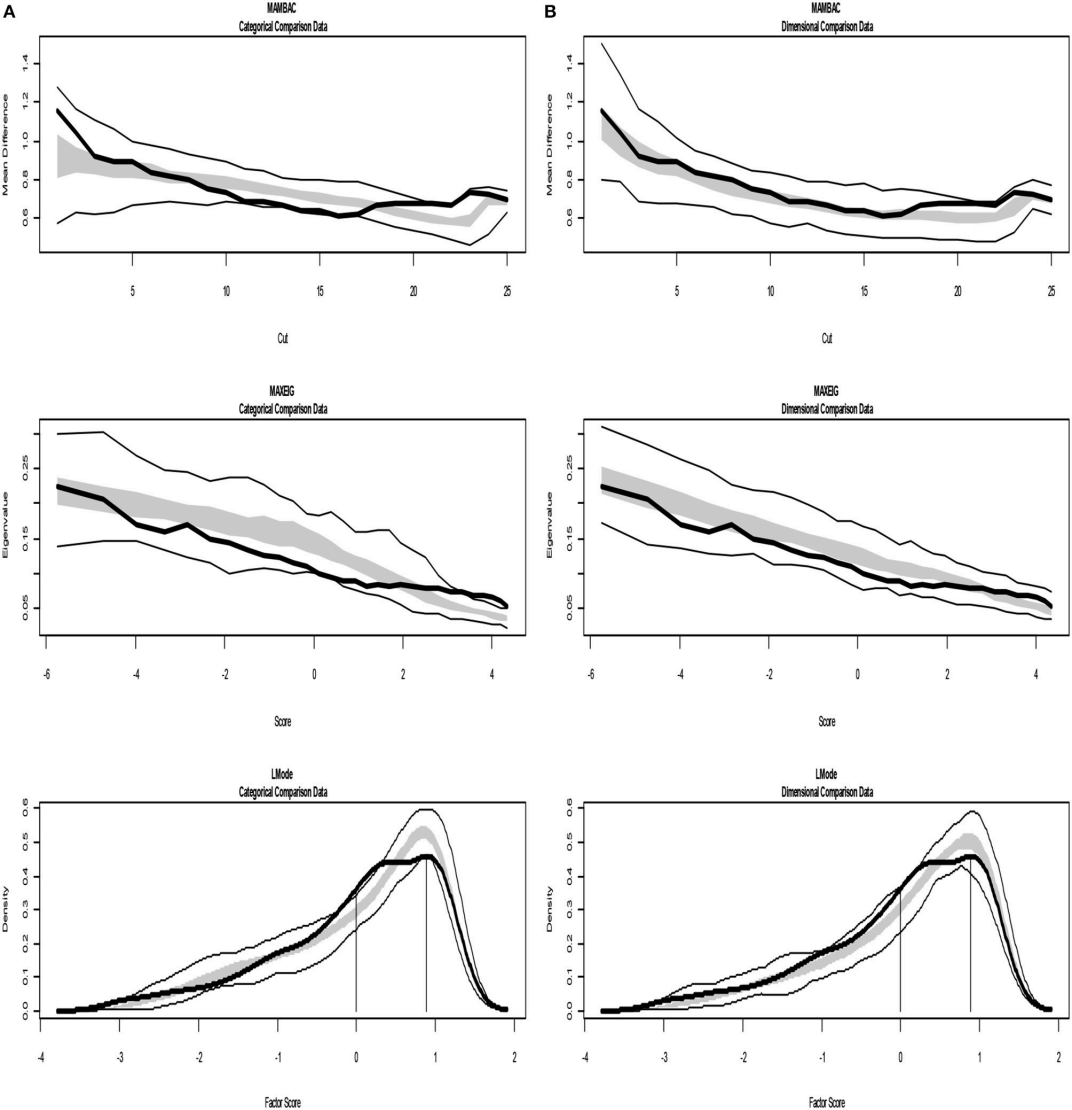
Input: Empirical data –study 3. **(A)** Comparison with categorical data. **(B)** Comparison with dimensional data. Results for MAMBAC (top), MAXEIG (middle), and L-Mode (bottom) analyses. Dark lines show the results for empirical data, and lighter lines show the results for parallel analyses of comparison data; the lines contain a band that spans ±1 SD from the mean at each data point on the curve.

The analyses of the empirical data are shown in Figures 6–8. Figure 6 shows the results from the Taxometric analysis using empirical data from study 1. Graphs of MAMBAC, MAXEIG, and L-Mode analyses of the real data are presented in contrast to idealized dimensional and categorical data sets. Visual inspection of MAMBAC (top), MAXEIG (middle), and L-Mode (bottom) curves do not provide evidences for taxon structures. Moreover, Table 4 depicts the values of the calculated CCFIs: MAMBAC = 0.571, MAXEIG-0.440, and L-Mode = 0.619. These CCFI values do not support any particular structure and the results are characterized as ambiguous. For study 2 the values of the calculated CCFIs are: MAMBAC = 0.460, MAXEIG-0.410, and L-Mode = 0.344 (Table 4), while Figure 7 shows the results from of MAMBAC, MAXEIG and L-Mode analyses. These results are also ambiguous as in study 1 providing no support for any particular structure.

**Table 4:** Comparison Curve Fit Index (CCFI) for artificial date and the empirical data from the three studies.

	**Artificial Data**		**Empirical Data**			
	**Continuous**	**Categorical -taxons**	**Study 1**	**Study 2**	**Study 3**
MAMBAC	0.341	0.920	0.571	0.460	0.340
MAXEIG	0.275	0.924	0.440	0.410	0.386
L-Mode	0.182	0.857	0.619	0.344	0.392
Mean	0.266	0.900	0.544	0.405	0.373

For study 3 the calculated values for CCFIs are: MAMBAC = 0.340, MAXEIG-0.386, and L-Mode = 0.392 (Table 4), while Figure 8 shows the results from of MAMBAC, MAXEIG and L-Mode analyses. The results slightly support the dimensional structure.

### Commentary of taxometric analysis

The nature of conclusions in the present endeavor is specified by the types of research hypotheses posited for this data-analytic technique. The taxometric inferential framework has been the cause of disparity and disagreement among scholars, since it is based on the consistency among multiple mathematical procedures rather than on a formal null hypothesis (Ho) (Ruscio, 2007). Inferential frameworks for taxometrics have been proposed with a focus on the detection of taxonic structure or on viewing dimensional structure as the null hypothesis. These demonstrated shortcomings and weak points, the main of which concern confirmation biases (Ruscio, 2007; Ruscio et al., 2011). The present taxometric analysis follows the logic of treating the hypothesized categorical and dimensional structural models as two competing hypotheses and seeks to evaluate their relative support. The crucial points to examine in this evaluation are the distinct features across taxonic and continuous data when other significant characteristics, such as sample size, number of indicators, and correlations are held constant. Monte Carlo techniques and the use of simulated comparison data serve these purposes and can provide support for one model against the other (Ruscio and Kaczetow, 2009).

## Discussion

The objectives of this paper were not merely to present the two psychometric methods conjointly, but to make a contribution in the field of inquiry probing children's mental representations, where research questions are open in an enduring debate for more than three decades. The nature of children's mental model and the role of psychometric predictors, are crucial issues, for which state-of-the-art and specialized methodologies are demanded for building contemporary theories for learning and development, and what is more for the pedagogical practices.

The first part of the present work illustrates the step-wise LCA in conjunction with the BCH and ML correction methods, providing improved unbiased estimations of the effects and standard deviations of external variables/predictors on the class membership. LCA has already been fruitfully used in children's mental models research (e.g., Straatemeier et al., 2008; Schneider and Hardy, 2013; Stamovlasis et al., 2013; Pluess et al., 2018), while with the advantages of the step-wise version, along with the improved correction methods for the parameters associated with external variables, becomes a valuable asset in this field of inquiry. In research for latent structures, a complete psychometrics enterprise would include the examination and fit comparisons with alternative latent structure models. However, this does not guarantee a secure choice; it is known that many continuous-variable models have statistically equivalent categorical or mixture alternatives (e.g., Halpin et al., 2011). In other words, fitting a LC model to empirical data does not imply that the latent construct under study is categorical, since a continuous model can also fit the data (Molenaar and Von Eye, 1994; Erosheva, 2005). In this work the choice of the latent structure modeling was driven by the underlying theories of mental models, which advocate a categorical construct. In LCA, the uncertainty in the anticipated taxonomy is sourcing out, not only from classification errors, but also from the ontological status of the latent variables under investigation (Borsboom et al., 2003). Thus, coupling latent structure models with taxometrics becomes essential strategy in psychometric endeavors for evaluation end interpretation of empirical data (McGrath and Walters, 2012). Moreover, the criteria mentioned earlier for applying TA (Lenzenweger, 2004) are definitely met, and the *taxon* hypothesis becomes a fundamental issue, because, besides its theoretical value it is highly related to pedagogical and educational practices (Taber, 2009). Taxometrics in this study provided ambiguous results about the structural model type. Despite this fact, the implementation of LCA is not a controversy, because theoretically, the latent variable in question embraces at least two classes: one is the eventually attained latent mental model, if happened, which is in line with the science view on the physical phenomena under study. Thus, LCA with covariates, as illustrated here, conserves its merit to mental model research, because the unbiased estimation of parameters related to external variables/predictors is a crucial issue for explaining learning-phenomena and developing theories (e.g., Theory of Mind, Wellman, 1992).

The ambiguous findings of taxometric analysis, nevertheless, raises a problematization, which also exists in psychometrics applied to current psychiatric research (Cramer et al., 2010, 2012a,b). This concerns the possibility that a latent construct functions as categorical entity for some individuals, while it could function as continuous for some others, contrary to the traditional assumption that for all cases latent constructs are either categorical or dimentinal. This has become an issue for discussion, especially with the recent development of network models, applied to psychological and psychiatric constructs (Borsboom and Cramer, 2013; De Schryver et al., 2015; Pe et al., 2015; Epskamp et al., 2017). It was pointed out, that for some individuals a disorder might appear as sudden transition in a categorical latent landscape, while for some others appears as gradual linear process (Borsboom and Cramer, 2013; Epskamp et al., 2017). It is worth noticing that the network analysis of psychological attributes had also a direct impact on methodological and epistemological issues (e.g., Borsboom, 2017; Guyon et al., 2017).

Returning to the present inquiry, analogous reflection could be made; it is reasonable to hypothesize that conceptual change might occur in both linear and nonlinear modes. That is, the latent variable under study could be continuous for some child and categorical for another. The hypothetical mental models driving children's responses are assumed to be the common cause for a number of observed response patterns. Let's deliberate this issue under the lens of network models. Considering two indicators, e.g., two items, the first on understanding the structure of matter and the other on understanding a physical property of matter, respectively. In reality they co-vary due to their common dependence on the hypothetical coherent and stable mental model:

**Figure:**
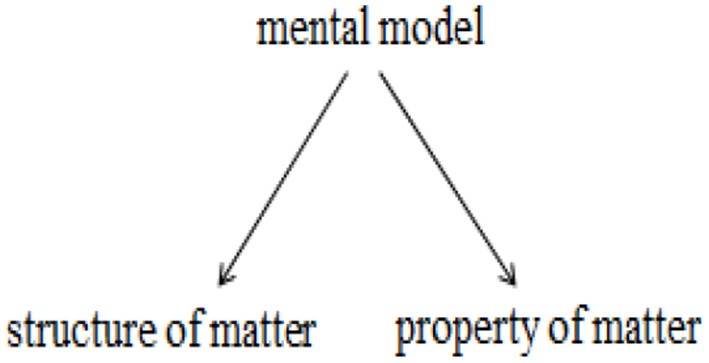


However, it is reasonable to assume that they could be causally related, in the sense that if one knows about the structure of matter, then he/she can deduce or infer its properties (Stamovlasis et al., 2012).

structure of matter → property of matter

Correlations and/or causal relationships among indicators and group of indicators can be depicted in a concept map, where pieces of knowledge are inter-connected portraying a network constellations of meaning. Such conjectural networks do not seem to reflect a localized latent variable that functions as psychological common causes. On the other hand, the individual nodes and connections in those networks may be influenced by cognitive factors, such as formal reasoning, field dependence /independence or divergent thinking. Individual differences in network structures, qualitatively and quantitatively may lead to different patterns in mental model representation and different modes and rates of change (conceptual change). For instance, weakly connected misconception (networks of meaning) might be easily altered by changing some node or edge in order to accommodate new information. On the contrary, strongly connected networks might behave differently: they can show strongly nonlinear behavior with sudden jumps from one state to another (Thom, 1975; van der Maas and Molenaar, 1992; Molenaar, 2004; Molenaar and Campbell, 2009; Stamovlasis, 2016).

The differences in network behavior and the underlying dynamics of different network structures are very relevant to the current discussion on the nature of mental representations, which is reduced to the distributional form of the latent variable in question, i.e., the kinds versus continua latent entities. Mental models may be discrete kinds for some children if their mental networks are strongly connected (coherent), while for some other individuals with weakly connected networks, they might be dimensional structures. That is, in empirical research studying inter-individual variability by employing collective data, even though continuous distributions are hypothesized and used, some intra–individual changes might be occurring as transition from a “naïve mental model” to the “scientific model.”

## Ethics statement

This study was carried out in accordance with the recommendations of ethics committee of Greek Ministry of Education which follows the guidelines of the American Psychological Association for treatment of human participants, that includes a written informed consent obtained from the parents or legal guardians of all children participants, oral assent of the children prior to test administration and approval by the school authorities. The research design was approved by the Institute of Educational Policy (Ministry of Education).

## Author contributions

All authors share equal contribution for all parts and steps of the study. DS conceptualized this study, and had a coordination role in organizing and writing the paper, being the corresponding author.

### Conflict of interest statement

The authors declare that the research was conducted in the absence of any commercial or financial relationships that could be construed as a potential conflict of interest.

## Appendix

**Appendix A:** Syntax and Coding in R for performing taxometric analysis via the RTaxometrics package^*^. An illustrative example (see also Supplementary Material for “Data sheet 1 and Presentation 1”).

# Load the RTaxometrics package
#(The input data is in MyData file)
> MyData < -read.table(″C:\\Users\\User\\Desktop\\R Taxo\\MyData.txt″, header = T)
> attach(MyData)
> names (MyData)
[1] ″S1″ ″S2″ ″S3″ ″S4″ ″S5″ ″S6″ ″S7″ ″S8″ ″S9″ # reads the input variables
>MyData # reads and types the input data
> x < -ClassifyCases(x, p = 0.5, cols = 1-9)
# Function preparing the data for taxometric analysis. It assigns cases to groups using the base-rate classification technique (x = input data matrix, p = base-rates used for classification, cols = columns containing data)
> CheckData(x) # function checking the suitability of the input empirical data for taxometric analysis,
# the output provides the relevant information (distributional characteristics, Cohen's d, within-group correlations etc.)
>test.dim < -CreateData(″dim″) # creates prototypical dimensional data
> test.cat < -CreateData(″cat″) # creates prototypical categorical data
# RunTaxometric analysis that includes all functions.
> RunTaxometrics(x, seed = 1,n.pop = 100000, n.samples = 100, reps = 10, MAMBAC = TRUE, assign.MAMBAC = 2, n.cuts = 25, n.end = 25, MAXEIG = TRUE, assign.MAXEIG = 3,windows = 30, LMode = TRUE, mode.l = −0.001, mode.r = 0.001,MAXSLOPE = TRUE)

* See also in the supplementary material with RTaxometrics-short tutorial and for further details in Ruscio (2017).
