# Supplementary material for: Illustration of Step-Wise Latent Class Modeling With Covariates and Taxometric Analysis in Research Probing Children's Mental Models in Learning Sciences
Source: Front Psychol. 2018 Apr 16;9:532. doi: 10.3389/fpsyg.2018.00532 (PMC5911829; doi:10.3389/fpsyg.2018.00532)
Supplement: Supplementary file 1 [file Presentation1.PDF]

Taxometrics in R–*a short tutorial*

By Dimitrios Stamovlasis, PhD

Syntax and Coding in R for performing taxometric analysis via the RTaxometrics package. (Detailed presentation could be found in Ruscio (2017).

Note: In a usual laptop with a moderate computational power these calculations may take about 40 min.

Part 1: Run taxometric with taxonic artificial data as input.

Load the RTaxometrics package

```
> test.cat<-CreateData("cat")   # creates prototypical categorical data
```

RunTaxometric analysis that includes all functions.

```
> RunTaxometrics(test.cat,seed=1,n.pop=100000, n.samples=100, reps=10,
MAMBAC=TRUE, assign.MAMBAC= 2, n.cuts=25, n.end=25, MAXEIG=TRUE,
assign.MAXEIG=3,windows=30, LMode=TRUE, mode.l=-0.001,
mode.r=0.001,MAXSLOPE=TRUE)
```

Explanations for the arguments:

x=the input data; seed= random number seed, the default is 1; n.pop= size of the finite populations of comparison data, the default is 100000; n.samples= the number of comparison data sets of each structure to generate and analyze, the default is 100; reps= the number of times to redo calculations if ties exist in the data, the default is 10; MAMBAC= command to perform MAMBAC procedure; The default TRUE; assign.MAMBAC=2, means that the input is the sum of the variables, while 1 means that all variable pairing can be assigned as input and output variables; n.cuts= the number of 'cuts' made along the input variable, the default is 25; n.end =the number of cases set apart at each extreme along the input variable, the default is 25; MAXEIG=command to perform MAXEIG, the default is TRUE if the number of variables k is greater or equal 3 and FALSE otherwise; assign.MAXEIG =3 means that the input is the sum of the variables, while 1 means that all variable pairing can be assigned as input and output variables and 2 means that each variable might be used as input once, the default is 1; windows= the number of overlapping windows use in MAXEIG analysis with default value of 50; overlap= the amount of overlap between windows in MAXEIG calculations, with default value of .90; LMode= the command to perform L-Mode analysis, the default is TRUE when k is equal or greater than 3 and FALSE otherwise; mode.l= left mode position sought by L-Mode procedure, with default -.001; mode.r= right mode position sought by L-Mode procedure.

*Further detailed presentation could be found in Ruscio (2017).

R starts computing

The output appears gradually as calculation go on.

```
STATUS OF PROGRAM EXECUTION
Checking for missing data
Checking classification variable
Checking for variance
Checking program parameters
  * no tied scores, reps set to 1
```

* more than 2 variables, MAXSLOPE will not be performed
Generating population of dimensional comparison data
Generating population of categorical comparison data
Generating taxon
Generating complement
Analyzing empirical data
Analyzing samples of dimensional comparison data
Analyzing samples of categorical comparison data

TAXOMETRIC ANALYSIS RESULTS

Summary of shared analytic specifications
sample size: 600
number of variables: 4
comparison data population size: 1e+05
comparison data samples: 100
comparison data taxon base rate: 0.5
replications: 1

Summary of MAMBAC analytic specifications
cuts: 25 evenly-spaced cuts beginning 25 cases from either extreme
indicators: output = one variable, input = sum of remaining variables
number of curves: 4

Summary of MAXEIG analytic specifications
subsamples: 30 windows that overlap 0.9
indicators: output = two variables, input = sum of remaining variables
number of curves: 6

Summary of L-Mode analytic specifications
position beyond which to search for left mode: -0.001
position beyond which to search for right mode: 0.001

Comparison Curve Fit Index (CCFI)
MAMBAC: 0.924
MAXEIG: 0.92
L-Mode: 0.857
mean: 0.9

Note: CCFI values can range from 0 (dimensional) to 1 (categorical).
The further a CCFI is from .50, the stronger the result.

Base Rate Estimates:
MAMBAC: 0.55
MAXEIG: 0.435
L-Mode:
based on location of left mode: 0.442
based on location of right mode: 0.599
mean: 0.521
mean: 0.502

Note: There is no evidence-based way to use base rate estimates to differentiate categorical and dimensional data. They should only be used if evidence **supports categorical structure**.
>

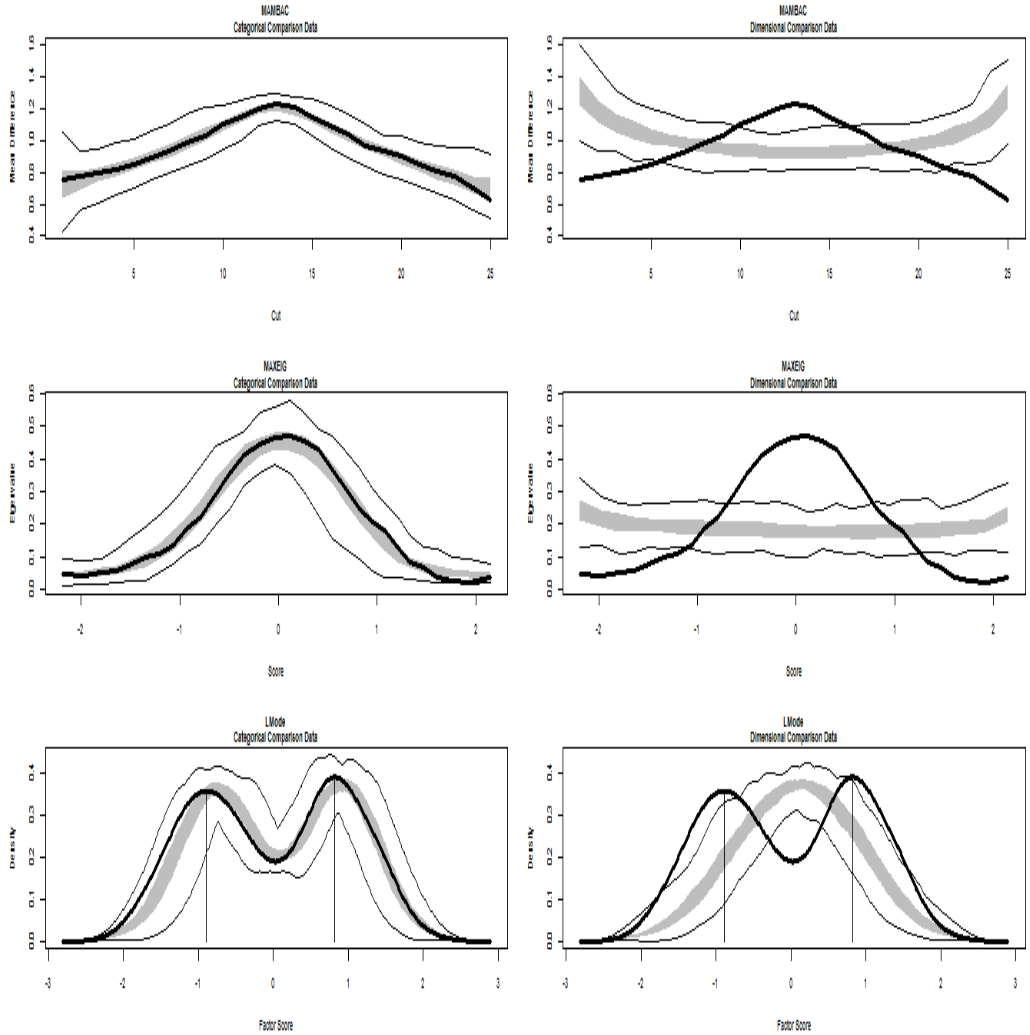

Taxometric Plots

Part 2: Run taxometric with empirical data (Study 3, Data=Earth04) as input.

Load the RTaxometrics package
#(The input data is in MyData file)

> Earth04<-read.table("C:\\Users\\User\\Desktop\\R Taxo\\Earth04.txt", header=T)

> names(Earth04)

[1] "Q1" "Q2" "Q3" "Q4" "Q5" "Q6" "Q7" "Q8" "Q9"

> Earth04

Q1 Q2 Q3 Q4 Q5 Q6 Q7 Q8 Q9

> CheckData(Earth04)

Sample size: N = 502
Taxon base rate: P = 0.06772908
  * This is smaller than the recommended minimum of P = .10.
Taxon size: n = 34
  * It may be difficult to differentiate categories when one is this small.
Complement size: n = 209
Number of variables: k = 8

Distributions:

	M	SD	Skewness	Kurtosis
v1	3.89	0.49	-4.48	19.48
v2	3.40	0.99	-1.43	0.66
v3	3.36	0.92	-1.28	0.53
v4	3.17	1.03	-1.08	-0.05
v5	3.28	0.99	-1.18	0.17
v6	2.75	1.41	-0.34	-1.80
v7	3.39	1.01	-1.43	0.64
v8	3.86	0.53	-3.57	11.48

Validities:

	Cohen's d
v1	0.18
v2	0.00
v3	0.09
v4	0.01
v5	0.15
v6	0.43
v7	0.10
v8	0.00
Mean	0.12

  * One or more values below the recommended minimum of d = 1.25.

Within-group correlations (taxon):

	v1	v2	v3	v4	v5	v6	v7	v8
v1	1.00	0.23	0.28	0.23	-0.16	0.17	0.26	0.56
v2	0.23	1.00	0.71	0.30	0.63	0.19	0.06	0.06
v3	0.28	0.71	1.00	0.40	0.52	0.23	0.28	0.23
v4	0.23	0.30	0.40	1.00	0.56	-0.11	0.18	-0.14
v5	-0.16	0.63	0.52	0.56	1.00	-0.03	-0.07	-0.07
v6	0.17	0.19	0.23	-0.11	-0.03	1.00	-0.18	0.08
v7	0.26	0.06	0.28	0.18	-0.07	-0.18	1.00	0.09
v8	0.56	0.06	0.23	-0.14	-0.07	0.08	0.09	1.00

Mean = 0.2
 * One or more values above the recommended maximum of r = .30.

Within-group correlations (complement):

	v1	v2	v3	v4	v5	v6	v7	v8
v1	1.00	0.08	0.23	0.05	0.12	0.05	-0.04	0.36
v2	0.08	1.00	0.59	0.24	0.52	0.12	0.21	0.16
v3	0.23	0.59	1.00	0.27	0.61	0.05	0.21	0.22
v4	0.05	0.24	0.27	1.00	0.39	0.03	0.30	-0.01
v5	0.12	0.52	0.61	0.39	1.00	0.16	0.19	0.19
v6	0.05	0.12	0.05	0.03	0.16	1.00	0.03	0.02
v7	-0.04	0.21	0.21	0.30	0.19	0.03	1.00	0.00
v8	0.36	0.16	0.22	-0.01	0.19	0.02	0.00	1.00

Mean = 0.19
 * One or more values above the recommended maximum of r = .30.

> x<-ClassifyCases(Earth04, p=0.5, cols=1-9)

Function preparing the data for taxometric analysis. It assigns cases to groups using the base-rate classification technique (x= input data matrix, p=base-rates used for classification, cols =columns containing data)

> x

	Q1	Q2	Q3	Q4	Q5	Q6	Q7	Q8	Q9	y[, last.col - 2]
1	4	4	3	3	3	1	3	4	1	1
2	4	2	2	1	2	1	4	4	1	1
3	4	3	2	3	3	1	4	4	1	1
4	1	2	3	3	3	1	4	3	3	1
5	2	4	3	1	3	2	4	4	1	1
6	2	1	1	4	1	1	4	4	1	1
7	4	1	1	3	1	1	4	4	1	1
8	4	1	1	4	1	1	4	4	1	1
9	4	3	4	3	4	4	4	4	1	2
10	4	4	4	4	4	1	4	4	1	1
11	4	3	3	4	3	1	4	4	1	1
12	4	3	4	3	3	2	4	4	2	1
13	4	2	2	3	2	2	2	4	2	1
14	4	4	3	4	4	4	4	4	1	2
15	4	1	1	4	4	1	1	4	1	1
16	4	1	1	1	1	1	1	4	1	1
17	4	3	3	3	4	4	4	4	1	1
18	2	3	3	1	3	1	4	4	4	1
19	4	4	4	3	4	4	3	4	4	2
20	4	4	4	4	4	1	3	4	3	2
21	4	1	1	4	1	1	4	4	1	1
22	4	4	3	4	3	4	4	4	4	2
23	4	4	4	4	4	4	4	4	1	2

24	4	4	4	4	3	1	4	4	1	1
25	4	4	4	3	3	1	3	4	3	1
26	4	4	2	1	2	1	4	2	3	1
27	4	4	4	4	4	4	4	4	4	2
28	4	4	4	4	4	4	4	4	3	2
29	4	4	4	3	3	2	4	4	4	2
30	4	1	1	1	1	3	1	4	1	1
31	4	4	4	3	3	1	1	4	1	1
32	4	4	4	4	4	4	4	4	1	2
33	4	4	3	3	3	4	4	4	3	2
34	4	3	4	4	2	4	4	4	3	2
35	4	4	4	3	3	2	4	4	3	2
36	4	4	4	3	4	4	3	4	4	2
37	4	3	3	4	3	4	4	4	4	2
38	4	3	3	1	3	3	2	4	3	1
39	4	3	3	4	3	4	1	4	4	1
40	4	3	3	4	3	4	4	4	4	2
41	4	2	2	1	2	1	4	4	1	1
42	4	4	3	1	3	1	1	4	1	1
43	4	3	3	1	2	4	1	4	1	1
44	2	2	2	3	1	1	4	1	1	1
45	4	3	4	4	4	1	4	4	2	1
46	4	1	3	4	4	4	4	4	4	2
47	4	4	3	4	4	4	4	4	3	2
48	4	1	1	4	3	4	4	4	4	1
49	4	4	4	4	3	1	1	4	1	1
50	4	2	1	2	2	2	4	4	3	1
51	4	4	3	4	3	4	4	4	3	2
52	4	4	4	3	4	4	4	4	4	2
53	4	4	4	4	4	4	4	4	4	2
54	4	3	4	3	3	3	4	4	3	2
55	4	4	4	4	4	4	4	4	3	2
56	4	3	3	3	4	4	3	4	4	2
57	4	4	4	3	4	1	4	4	1	1
58	4	4	3	3	4	1	4	4	1	1
59	4	2	2	3	2	1	4	4	2	1
60	4	4	4	1	4	1	4	4	3	1
61	4	4	4	3	4	1	4	4	2	1
62	4	4	4	3	4	4	3	4	2	2
63	4	4	3	3	4	4	4	4	1	2
64	4	3	4	4	4	4	4	4	1	2
65	4	1	4	1	1	1	4	4	1	1
66	4	4	3	3	1	1	3	4	1	1
67	4	3	2	3	2	4	3	4	1	1
68	4	3	3	4	4	4	4	4	4	2
69	4	1	1	1	1	3	4	4	4	1
70	4	4	2	3	2	4	2	4	1	1
71	4	4	4	3	4	4	2	4	2	2

72	4	3	3	3	3	3	4	4	2	1
73	4	4	4	3	4	4	4	4	3	2
74	4	3	3	4	3	4	4	2	2	2
75	4	4	4	3	4	4	4	4	1	2
76	4	2	4	3	2	4	4	4	3	1
77	4	4	4	4	4	4	4	4	4	2
78	4	4	3	3	4	4	1	4	1	1
79	4	4	4	3	4	4	4	4	3	2
80	4	3	4	3	4	3	3	4	3	2
81	4	4	4	3	4	4	4	4	4	2
82	4	2	4	2	2	4	2	2	4	1
83	4	4	4	3	4	4	4	4	3	2
84	4	2	2	1	2	4	2	2	1	1
85	4	1	1	1	1	1	1	4	1	1
86	4	3	4	3	3	3	4	4	3	2
87	4	4	4	3	4	4	4	4	3	2
88	4	4	4	3	4	4	4	4	3	2
89	4	4	4	4	4	4	4	4	4	2
90	4	4	4	4	3	1	4	4	1	1
91	4	4	3	3	4	4	4	4	1	2
92	4	4	4	4	4	4	4	4	4	2
93	4	4	3	3	4	1	1	4	4	1
94	4	4	4	3	4	4	4	4	3	2
95	4	1	1	1	1	4	1	4	3	1
96	1	1	1	1	1	4	1	4	3	1
97	4	1	1	1	1	4	1	4	3	1
98	4	4	2	3	4	4	4	4	3	2
99	4	4	4	3	4	4	3	2	3	2
100	4	4	4	4	4	4	4	4	4	2
101	4	1	3	3	3	1	4	4	1	1
102	2	2	2	2	2	4	2	2	3	1
103	4	4	4	2	2	1	1	4	1	1
104	4	4	2	4	4	4	4	4	3	2
105	2	2	2	2	2	1	2	2	1	1
106	4	4	3	4	3	4	4	2	4	2
107	4	4	2	4	4	4	4	4	1	2
108	4	4	4	4	4	4	4	4	4	2
109	4	3	4	4	4	4	3	4	4	2
110	4	4	4	3	4	1	4	4	2	1
111	4	4	4	3	4	4	4	4	4	2
112	2	2	4	2	4	1	4	2	1	1
113	4	4	4	3	4	4	2	4	2	2
114	4	4	4	4	4	4	4	4	4	2
115	4	4	4	4	4	4	4	4	4	2
116	4	1	4	4	1	4	4	4	4	1
117	4	4	4	4	4	4	4	2	4	2
118	4	4	4	4	4	4	4	4	4	2
119	4	4	4	4	4	1	3	4	4	2

120	4	4	4	4	4	4	4	4	4	2
121	4	4	4	4	4	4	4	4	4	2
122	4	4	4	3	3	4	3	2	1	1
123	4	4	4	4	4	4	4	4	4	2
124	4	4	4	4	4	3	4	4	4	2
125	4	4	4	4	4	4	4	4	4	2
126	4	4	4	4	4	4	4	4	4	2
127	4	4	4	4	4	4	4	4	4	2
128	4	4	4	4	4	4	4	4	4	2
129	4	4	4	4	4	4	4	4	4	2
130	4	1	1	1	4	4	4	4	4	1
131	4	4	4	4	4	4	4	4	4	2
132	4	4	4	4	4	4	4	4	4	2
133	4	4	4	4	4	4	4	4	4	2
134	4	4	4	4	4	4	4	4	4	2
135	4	4	4	4	4	4	4	4	3	2
136	4	4	4	3	4	4	4	4	4	2
137	4	4	4	4	4	4	4	4	4	2
138	4	4	4	3	4	4	4	4	3	2
139	4	2	4	4	2	4	4	4	3	2
140	4	4	4	4	4	4	4	4	4	2
141	4	4	4	4	4	1	4	4	4	2
142	4	4	4	1	3	4	2	4	1	1
143	4	4	4	4	4	4	4	4	4	2
144	2	2	2	1	1	4	2	4	3	1
145	4	4	4	4	4	4	4	4	3	2
146	4	4	2	2	4	4	2	4	3	1
147	4	4	4	4	4	4	4	4	3	2
148	4	4	4	4	4	4	4	4	3	2
149	4	4	4	4	4	4	4	4	4	2
150	4	4	2	3	3	4	4	4	3	2
151	4	4	4	4	4	1	4	4	4	2
152	2	4	4	4	4	4	4	4	1	2
153	4	4	4	4	4	4	4	2	4	2
154	4	4	4	4	4	4	4	4	4	2
155	4	2	4	4	2	2	2	4	4	1
156	4	4	4	4	4	4	4	4	4	2
157	4	4	4	1	3	4	4	2	3	2
158	4	4	4	4	4	4	4	4	4	2
159	4	4	4	3	4	4	4	4	3	2
160	4	4	4	3	4	4	2	4	4	2
161	4	4	4	4	4	4	3	4	3	2
162	4	4	4	4	4	4	4	4	4	2
163	4	4	4	4	4	4	4	2	3	2
164	4	4	4	3	4	4	4	4	4	2
165	4	4	4	4	4	4	4	4	4	2
166	4	4	4	4	3	1	3	4	1	1
167	4	4	4	3	2	4	2	4	4	2

168	4	4	4	4	4	4	4	4	4	2
169	4	4	2	3	2	4	3	4	4	1
170	4	4	4	4	4	1	1	4	1	1
171	2	2	2	2	4	1	2	2	2	1
172	4	2	3	4	3	1	2	4	1	1
173	4	4	2	2	4	4	4	4	3	2
174	4	4	4	3	4	4	3	4	4	2
175	4	4	4	4	4	1	4	4	1	1
176	4	2	2	2	2	2	1	2	1	1
177	2	2	1	2	2	1	2	2	1	1
178	4	4	3	2	4	2	4	4	3	1
179	4	4	4	4	4	1	3	4	4	2
180	4	2	3	1	1	1	1	4	1	1
181	4	4	2	4	2	4	2	4	3	1
182	4	4	2	3	2	4	2	4	4	1
183	4	3	3	3	3	2	4	4	4	1
184	4	1	4	1	4	4	1	4	1	1
185	4	4	3	4	4	1	4	4	1	1
186	4	1	3	4	3	2	4	4	1	1
187	1	4	2	4	4	4	1	4	1	1
188	4	1	3	4	1	1	4	4	1	1
189	4	3	3	3	3	1	3	4	1	1
190	4	4	3	4	3	1	4	4	1	1
191	4	4	4	4	4	3	4	4	3	2
192	4	3	3	3	3	1	2	4	1	1
193	4	4	3	3	3	1	1	4	1	1
194	4	3	4	1	1	1	1	4	1	1
195	4	4	4	1	4	1	4	4	1	1
196	4	2	3	4	2	4	4	4	4	2
197	4	2	3	4	4	4	4	4	4	2
198	4	2	3	4	4	1	4	4	2	1
199	4	4	3	4	4	1	4	2	1	1
200	4	4	4	3	3	1	4	4	1	1
201	4	1	4	2	3	1	2	4	1	1
202	4	1	1	1	1	1	1	4	1	1
203	4	3	3	4	3	1	4	4	1	1
204	4	4	4	3	4	1	4	4	4	2
205	4	4	3	3	3	1	3	4	1	1
206	4	4	4	3	4	1	3	4	2	1
207	4	4	3	4	3	1	3	4	2	1
208	4	4	3	3	3	1	3	4	1	1
209	4	4	4	4	4	1	4	4	1	1
210	4	4	4	3	3	4	4	4	2	2
211	4	4	4	3	3	1	4	4	1	1
212	4	4	4	3	4	4	4	4	4	2
213	4	3	4	3	3	1	3	4	1	1
214	4	2	4	3	2	4	3	4	3	1
215	4	1	1	1	1	4	4	4	1	1

216	2	4	4	4	4	2	4	2	1	1
217	4	3	4	3	4	3	4	4	1	1
218	4	2	2	3	3	1	3	4	1	1
219	4	4	2	2	2	1	2	4	1	1
220	4	4	4	3	4	4	3	4	3	2
221	4	4	3	3	4	4	3	4	3	2
222	4	4	2	1	4	4	4	4	4	2
223	4	2	2	2	2	4	2	4	2	1
224	4	3	2	4	2	4	4	4	1	1
225	4	4	4	4	4	1	4	4	1	1
226	4	2	2	2	2	4	2	4	2	1
227	4	3	3	1	3	4	1	4	3	1
228	4	4	4	3	4	4	4	4	4	2
229	4	4	4	1	4	4	4	4	4	2
230	4	4	3	3	4	2	1	4	2	1
231	4	4	4	1	4	1	2	4	1	1
232	4	4	2	1	4	1	2	4	1	1
233	4	4	3	3	4	1	4	4	1	1
234	4	4	4	4	3	4	3	4	1	2
235	4	4	3	4	3	4	4	4	1	2
236	4	4	3	4	3	1	4	4	1	1
237	4	4	4	4	4	4	4	4	1	2
238	4	3	3	2	3	4	4	4	3	1
239	2	3	1	1	2	1	4	4	1	1
240	4	4	4	4	4	4	4	4	4	2
241	4	3	4	3	3	1	3	4	1	1
242	4	4	4	3	4	4	4	4	4	2
243	4	1	4	4	3	4	3	4	4	2
244	4	4	4	4	4	4	4	4	3	2
245	4	3	4	1	3	1	1	4	1	1
246	4	3	3	4	3	4	4	4	4	2
247	4	4	4	4	4	4	4	4	4	2
248	4	3	4	4	4	1	4	4	1	1
249	4	4	4	4	3	1	1	4	1	1
250	4	4	3	3	4	1	4	4	1	1
251	4	3	3	3	3	1	4	4	1	1
252	4	4	3	2	3	1	4	4	1	1
253	4	4	4	1	4	4	4	4	4	2
254	4	4	4	3	4	4	4	4	4	2
255	4	4	4	4	4	4	4	4	3	2
256	4	1	1	1	1	4	1	4	3	1
257	4	4	2	3	4	1	4	4	4	1
258	4	4	4	3	4	3	3	4	1	1
259	4	4	3	4	4	3	4	4	3	2
260	4	4	4	4	4	1	4	4	4	2
261	4	4	4	3	4	1	4	4	1	1
262	4	2	2	1	1	1	4	4	2	1
263	4	4	4	1	4	1	1	4	4	1

264	4	4	4	3	4	4	4	4	4	2
265	4	4	2	1	1	1	4	4	1	1
266	4	4	3	4	4	4	4	2	1	2
267	4	3	4	4	4	4	4	4	4	2
268	4	4	4	4	4	4	4	4	4	2
269	4	4	4	4	4	3	4	4	4	2
270	4	4	4	4	4	4	4	4	3	2
271	4	4	2	4	4	2	2	2	3	1
272	4	4	4	3	4	3	4	4	4	2
273	4	4	4	4	4	4	4	4	4	2
274	4	4	4	4	4	4	4	4	3	2
275	4	4	4	4	4	4	4	4	3	2
276	4	3	4	4	4	4	4	4	4	2
277	4	4	4	2	3	1	4	4	1	1
278	4	4	2	3	4	4	4	4	3	2
279	4	4	4	4	4	4	4	4	3	2
280	4	4	4	3	2	1	4	2	1	1
281	4	4	4	4	4	4	4	4	3	2
282	4	4	4	4	4	4	4	4	4	2
283	4	4	4	4	4	4	4	4	4	2
284	4	4	4	4	4	4	4	4	3	2
285	4	4	4	4	4	4	4	4	3	2
286	4	4	4	4	4	4	4	4	4	2
287	4	4	4	1	1	2	4	4	1	1
288	4	4	4	4	4	4	4	4	1	2
289	4	1	4	3	1	1	3	4	4	1
290	4	4	2	4	4	1	4	4	4	2
291	4	4	2	3	1	1	3	4	1	1
292	4	1	1	1	1	4	1	4	3	1
293	4	3	3	4	3	1	2	4	1	1
294	4	3	4	4	4	4	4	4	3	2
295	4	3	4	4	4	1	4	4	1	1
296	4	4	4	3	3	1	3	4	1	1
297	4	4	3	3	4	2	4	4	4	2
298	4	4	3	3	3	1	4	4	1	1
299	4	4	3	3	2	1	2	2	1	1
300	4	4	3	4	3	4	3	4	4	2
301	4	4	4	4	4	4	4	4	4	2
302	4	4	3	2	4	1	2	4	4	1
303	4	3	3	1	3	1	3	4	3	1
304	4	4	4	4	3	3	4	4	4	2
305	4	4	4	4	4	4	4	4	3	2
306	4	4	4	4	4	4	4	4	3	2
307	4	3	4	4	4	2	1	4	1	1
308	4	4	4	3	4	4	3	4	4	2
309	4	4	2	4	4	4	4	4	4	2
310	1	4	3	4	3	4	4	4	4	2
311	4	4	4	4	4	1	4	4	2	2

312	4	4	4	4	4	4	4	4	4	2
313	4	4	2	3	2	4	4	4	3	1
314	4	4	4	3	4	4	3	4	2	2
315	4	4	4	4	4	4	4	4	4	2
316	4	4	4	3	4	4	3	4	4	2
317	4	4	3	3	4	3	3	4	2	1
318	4	4	3	4	4	3	4	4	4	2
319	4	4	4	3	4	3	4	4	4	2
320	4	4	4	4	4	4	4	4	3	2
321	4	4	4	3	4	4	3	4	4	2
322	4	4	3	4	4	4	4	4	4	2
323	4	4	4	3	4	4	4	4	4	2
324	4	4	4	1	4	3	4	4	1	1
325	4	4	4	3	4	1	4	4	4	2
326	4	4	4	4	4	4	4	4	3	2
327	4	2	4	4	4	1	4	4	1	1
328	4	2	2	2	2	2	4	4	2	1
329	4	4	4	4	4	4	4	4	3	2
330	4	4	4	3	4	4	4	4	1	2
331	4	4	3	4	4	1	4	4	3	2
332	4	1	4	1	1	4	1	4	4	1
333	4	4	3	3	4	2	1	4	1	1
334	4	4	4	4	4	4	4	4	3	2
335	4	1	1	2	1	1	1	4	1	1
336	4	4	4	4	4	4	4	4	4	2
337	4	4	4	3	4	1	4	4	1	1
338	4	4	4	1	1	4	4	4	2	1
339	4	4	4	4	4	4	4	4	4	2
340	4	2	2	3	2	1	1	4	1	1
341	4	4	4	1	4	4	4	4	3	2
342	4	3	2	4	3	1	2	4	1	1
343	4	3	2	4	3	3	1	2	1	1
344	4	4	4	1	4	4	4	4	3	2
345	4	2	2	3	1	2	3	4	1	1
346	4	4	4	3	4	4	4	2	4	2
347	4	1	1	1	3	1	1	4	1	1
348	4	4	4	1	1	1	4	4	1	1
349	2	4	1	2	2	1	3	4	1	1
350	4	3	4	2	3	2	4	4	1	1
351	4	1	1	1	1	1	4	4	1	1
352	2	2	3	4	2	1	3	4	1	1
353	4	4	3	4	3	4	4	4	3	2
354	4	4	4	3	3	1	2	4	1	1
355	2	4	3	4	2	1	3	4	1	1
356	4	3	3	4	3	1	4	4	1	1
357	4	3	4	3	3	3	3	4	2	1
358	2	4	3	1	3	1	4	4	1	1
359	4	4	4	3	4	1	3	4	1	1

360	4	3	4	4	4	1	4	4	1	1
361	4	4	4	3	3	1	4	4	1	1
362	4	4	3	1	2	1	4	4	1	1
363	4	1	1	4	1	1	3	1	1	1
364	4	2	3	3	2	1	4	2	1	1
365	4	4	3	3	2	1	3	4	1	1
366	1	1	1	3	1	1	4	1	1	1
367	4	4	4	3	3	4	2	4	1	1
368	4	4	4	3	4	4	3	4	3	2
369	4	3	4	3	4	1	3	4	1	1
370	4	4	4	4	4	4	4	4	3	2
371	4	4	3	3	4	4	3	4	1	1
372	4	4	4	4	4	4	4	4	4	2
373	4	4	4	4	4	4	3	4	1	2
374	4	3	4	3	4	1	3	4	1	1
375	4	4	3	3	3	1	3	4	1	1
376	4	4	4	3	3	4	3	4	1	1
377	4	4	4	3	3	4	3	4	3	2
378	4	4	4	3	4	4	4	4	4	2
379	4	4	4	4	4	1	3	4	1	1
380	4	1	1	1	1	1	1	4	1	1
381	4	3	4	3	4	4	4	4	1	2
382	4	4	3	3	4	1	4	4	2	1
383	4	3	3	1	3	1	1	4	3	1
384	4	4	3	4	3	1	4	4	1	1
385	4	3	4	4	4	4	4	4	3	2
386	4	4	4	3	4	4	4	4	1	2
387	4	4	4	3	4	1	4	4	1	1
388	4	1	1	3	3	1	1	4	1	1
389	4	4	4	4	3	1	4	4	1	1
390	4	1	4	4	1	1	4	4	1	1
391	4	1	3	1	1	1	1	4	1	1
392	4	2	4	3	1	1	1	4	1	1
393	4	1	3	4	3	1	4	4	1	1
394	4	2	2	4	2	4	4	4	1	1
395	4	2	3	3	4	1	3	4	1	1
396	3	3	3	3	4	4	4	3	3	2
397	4	4	3	4	3	1	3	2	2	1
398	4	3	4	3	3	1	4	4	1	1
399	4	3	3	3	3	1	4	4	1	1
400	4	2	4	3	4	1	3	4	4	1
401	4	4	2	3	2	4	2	4	1	1
402	4	2	2	4	2	4	4	4	4	1
403	4	1	3	3	1	1	3	4	2	1
404	4	3	4	4	3	4	4	4	2	2
405	4	3	4	4	4	4	4	4	4	2
406	4	4	2	4	1	4	4	4	1	1
407	4	4	4	4	4	4	4	4	3	2

408	4	3	4	4	4	4	1	4	1	1
409	4	4	3	4	4	4	4	4	4	2
410	4	2	2	4	2	4	4	4	1	1
411	4	4	4	4	4	1	4	4	1	1
412	4	4	4	4	3	3	3	2	4	2
413	4	4	4	4	4	4	4	4	3	2
414	4	4	2	4	2	1	4	4	4	1
415	4	4	4	4	4	1	4	4	1	1
416	4	4	4	4	4	1	4	4	1	1
417	4	2	2	3	2	3	4	4	1	1
418	4	4	4	4	4	1	4	4	1	1
419	4	4	4	4	4	4	4	4	2	2
420	4	4	4	2	4	1	4	4	1	1
421	4	4	4	4	4	4	4	4	4	2
422	4	4	4	4	4	4	4	2	4	2
423	4	4	4	4	4	4	4	4	4	2
424	4	4	4	4	4	4	4	4	3	2
425	4	4	4	4	4	4	4	4	4	2
426	4	4	3	4	4	4	4	4	4	2
427	4	3	4	4	3	1	1	4	1	1
428	4	4	4	4	4	4	4	4	3	2
429	4	3	1	4	1	1	4	2	1	1
430	4	3	4	1	3	3	1	4	4	1
431	4	2	2	2	2	4	2	4	1	1
432	4	4	4	4	4	1	3	4	1	1
433	4	4	4	3	4	4	3	4	3	2
434	4	4	4	3	4	4	4	4	1	2
435	1	1	4	4	3	4	4	4	3	1
436	4	4	4	4	3	4	4	4	1	2
437	4	4	4	4	3	1	4	4	1	1
438	4	4	4	3	4	4	3	4	1	2
439	4	4	4	4	4	4	4	4	4	2
440	4	2	4	4	2	4	4	4	4	2
441	4	4	4	1	4	4	4	4	1	1
442	4	4	4	4	4	4	4	4	4	2
443	4	2	4	3	4	4	4	4	4	2
444	4	4	4	1	4	4	4	4	4	2
445	4	4	4	4	4	4	4	4	1	2
446	4	4	4	3	4	4	4	4	4	2
447	4	4	4	4	3	1	4	4	1	1
448	4	4	4	4	4	4	4	4	4	2
449	4	4	4	4	4	4	4	4	4	2
450	4	4	4	4	4	4	4	4	4	2
451	4	4	4	1	3	4	4	4	3	2
452	4	4	4	4	4	4	4	4	4	2
453	4	1	4	2	4	1	1	4	3	1
454	4	2	2	2	2	4	2	4	4	1
455	4	4	4	4	4	4	4	4	4	2

456	4	2	3	1	2	1	4	4	1	1
457	4	3	4	1	3	4	4	4	1	1
458	3	4	4	1	1	4	1	4	4	1
459	4	4	4	4	4	1	4	4	1	1
460	3	4	4	4	4	4	4	4	4	2
461	4	4	2	1	1	4	3	4	1	1
462	3	4	4	2	4	4	4	3	3	2
463	4	4	3	4	3	3	4	4	4	2
464	4	3	3	4	3	3	4	4	4	2
465	4	4	3	4	3	3	4	4	3	2
466	4	4	3	4	3	3	4	4	4	2
467	4	4	3	2	3	3	4	4	4	2
468	4	4	4	3	4	1	1	4	1	1
469	4	4	4	4	4	1	4	4	1	1
470	4	4	4	4	4	1	4	4	1	1
471	4	4	4	4	4	1	4	4	1	1
472	4	4	4	4	4	1	4	4	1	1
473	4	2	3	2	3	1	2	4	1	1
474	4	4	4	1	4	1	1	4	1	1
475	4	4	4	4	4	1	4	4	1	1
476	4	4	4	4	4	1	4	4	1	1
477	4	4	4	4	4	1	2	4	1	1
478	4	4	4	4	4	4	4	4	3	2
479	4	4	4	4	4	4	4	4	4	2
480	4	4	4	4	4	4	4	4	4	2
481	4	4	4	4	4	4	4	4	4	2
482	4	4	4	4	4	4	4	4	4	2
483	4	4	3	3	3	3	4	4	4	2
484	4	3	3	3	3	1	3	4	2	1
485	4	3	3	3	3	1	2	4	4	1
486	4	3	3	3	3	1	3	4	2	1
487	4	3	3	3	3	4	3	4	2	1
488	4	1	1	1	1	1	1	4	1	1
489	4	1	1	4	4	1	4	4	1	1
490	4	4	4	4	4	1	4	4	1	1
491	4	4	4	4	4	1	2	4	1	1
492	4	4	4	4	4	1	4	4	1	1
493	4	4	4	4	1	1	4	4	1	1
494	4	4	4	3	4	1	3	4	1	1
495	4	4	4	4	4	1	4	4	1	1
496	4	4	4	4	4	1	4	4	1	1
497	4	4	4	4	4	4	1	4	1	1
498	4	1	4	4	4	4	4	4	1	1
499	4	4	4	4	4	4	4	4	4	2
500	4	4	4	4	4	4	4	4	4	2
501	4	4	2	4	4	4	4	4	4	2
502	4	4	4	4	4	4	4	4	4	2

> CheckData(x)

Sample size: N = 502
Taxon base rate: P = 0.4800797
Taxon size: n = 241
Complement size: n = 261
Number of variables: k = 9

Distributions:

	M	SD	Skewness	Kurtosis
v1	3.89	0.49	-4.48	19.48
v2	3.40	0.99	-1.43	0.66
v3	3.36	0.92	-1.28	0.53
v4	3.17	1.03	-1.08	-0.05
v5	3.28	0.99	-1.18	0.17
v6	2.75	1.41	-0.34	-1.80
v7	3.39	1.01	-1.43	0.64
v8	3.86	0.53	-3.57	11.48
v9	2.42	1.31	0.05	-1.75

Validities:

	Cohen's d
v1	0.31
v2	0.97
v3	0.92
v4	0.86
v5	1.23
v6	1.93
v7	1.00
v8	0.16
v9	1.83
Mean	1.02

 * One or more values below the recommended minimum of d = 1.25.

Within-group correlations (taxon):

	v1	v2	v3	v4	v5	v6	v7	v8	v9
v1	1.00	-0.01	0.07	-0.01	0.06	-0.04	-0.05	0.05	0.05
v2	-0.01	1.00	0.10	-0.08	0.37	-0.06	0.01	-0.02	-0.03
v3	0.07	0.10	1.00	0.03	0.22	0.06	-0.11	0.03	0.04
v4	-0.01	-0.08	0.03	1.00	0.02	-0.03	0.17	0.05	0.08
v5	0.06	0.37	0.22	0.02	1.00	0.05	0.07	0.08	0.02
v6	-0.04	-0.06	0.06	-0.03	0.05	1.00	0.04	-0.05	-0.11
v7	-0.05	0.01	-0.11	0.17	0.07	0.04	1.00	0.01	0.08
v8	0.05	-0.02	0.03	0.05	0.08	-0.05	0.01	1.00	0.02
v9	0.05	-0.03	0.04	0.08	0.02	-0.11	0.08	0.02	1.00

Mean = 0.03
 * One or more values above the recommended maximum of r = .30.

```
Within-group correlations (complement):
     v1    v2    v3    v4    v5    v6    v7    v8    v9
v1  1.00  0.14  0.19  0.08  0.12 -0.04 -0.02  0.34 -0.06
v2  0.14  1.00  0.51  0.23  0.51 -0.16  0.17  0.10 -0.17
v3  0.19  0.51  1.00  0.26  0.53 -0.18  0.17  0.17 -0.13
v4  0.08  0.23  0.26  1.00  0.35 -0.20  0.31 -0.02 -0.20
v5  0.12  0.51  0.53  0.35  1.00 -0.20  0.14  0.13 -0.11
v6 -0.04 -0.16 -0.18 -0.20 -0.20  1.00 -0.20  0.02  0.37
v7 -0.02  0.17  0.17  0.31  0.14 -0.20  1.00  0.00 -0.13
v8  0.34  0.10  0.17 -0.02  0.13  0.02  0.00  1.00  0.02
v9 -0.06 -0.17 -0.13 -0.20 -0.11  0.37 -0.13  0.02  1.00
Mean = 0.09
  * One or more values above the recommended maximum of r = .30.
```

```
> RunTaxometrics(x,seed=1,n.pop=100000, n.samples=100, reps=10, MAMBAC=TRUE,
assign.MAMBAC= 2, n.cuts=25, n.end=25, MAXEIG=TRUE, assign.MAXEIG=3,windows=30,
LMode=TRUE, mode.l=-0.001, mode.r=0.001,MAXSLOPE=TRUE)
```

R starts computing

STATUS OF PROGRAM EXECUTION

```
Checking for missing data
Checking classification variable
Checking for variance
Checking program parameters
  * more than 2 variables, MAXSLOPE will not be performed
Generating population of dimensional comparison data
Generating population of categorical comparison data
  Generating taxon
  Generating complement
Analyzing empirical data
Analyzing samples of dimensional comparison data
Analyzing samples of categorical comparison data
```

TAXOMETRIC ANALYSIS RESULTS

```
Summary of shared analytic specifications
  sample size:  502
  number of variables:  9
  comparison data population size:  1e+05
  comparison data samples:  100
  comparison data taxon base rate:  0.48
  replications:  10

Summary of MAMBAC analytic specifications
  cuts:  25 evenly-spaced cuts beginning 25 cases from either extreme
```

indicators: output = one variable, input = sum of remaining variables
number of curves: 9

Summary of MAXEIG analytic specifications
  subsamples: 30 windows that overlap 0.9
  indicators: output = two variables, input = sum of remaining variables
  number of curves: 36

Summary of L-Mode analytic specifications
  position beyond which to search for left mode: -0.001
  position beyond which to search for right mode: 0.001

Comparison Curve Fit Index (CCFI)
  MAMBAC: 0.34
  MAXEIG: 0.386
  L-Mode: 0.392
  mean: 0.373

Note: CCFI values can range from 0 (dimensional) to 1 (categorical).
The further a CCFI is from .50, the stronger the result.

Base Rate Estimates:
  MAMBAC: 0.433
  MAXEIG: 0.855
  L-Mode:
    based on location of left mode: 0
    based on location of right mode: 0.561
    mean: 0.281
  mean: 0.523

Note: There is no evidence-based way to use base rate estimates to differentiate categorical and dimensional data. They should only be used if evidence **supports categorical** structure.

>

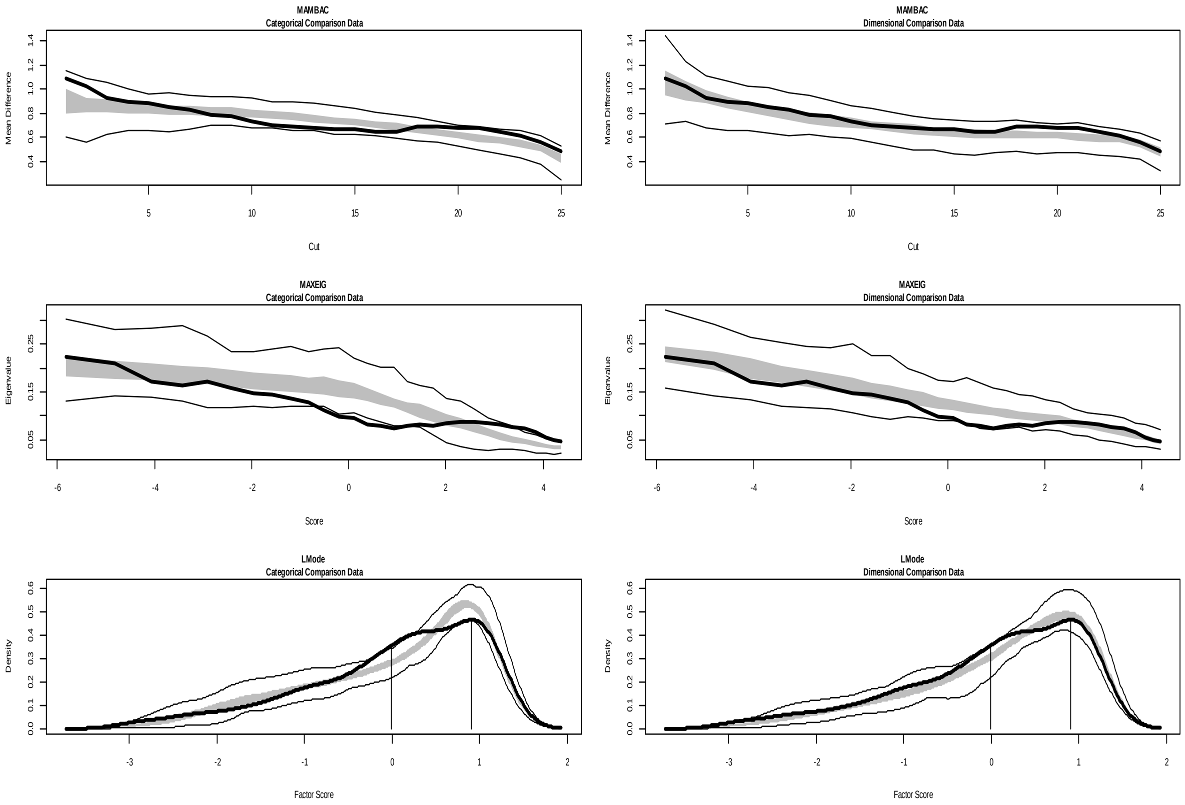

MAMBAC
Categorical Comparison Data
Mean Difference
Cut
MAMBAC
Dimensional Comparison Data
Mean Difference
Cut
MAXEIG
Categorical Comparison Data
Eigenvalue
Score
MAXEIG
Dimensional Comparison Data
Eigenvalue
Score
LMode
Categorical Comparison Data
Density
Factor Score
LMode
Dimensional Comparison Data
Density
Factor Score
